# Identification and characterization of trimeric autotransporter adhesins from *Photobacterium damselae* subsp. *piscicida*

**DOI:** 10.3389/fcimb.2026.1964857

**Published:** 2026-10-30

**Authors:** Johnny Lisboa, José Viveiros, Alexandra Teixeira, Inês Loureiro, Inês Lua Freitas, Gabriela Afonso, Bruno Cavadas, Pedro Oliveira, Jorge E. Azevedo, Nuno Miguel Simões dos Santos, Ana do Vale

**Affiliations:** 1 i3S - Instituto de Investigação e Inovação em Saúde, Universidade do Porto, Porto, Portugal; 2 Instituto de Biologia Molecular e Celular, Universidade do Porto, Porto, Portugal; 3 FCUP - Faculdade de Ciências, Universidade do Porto, Porto, Portugal; 4 ICBAS - Instituto de Ciências Biomédicas Abel Salazar, Universidade do Porto, Porto, Portugal; 5 Escola de Ciências da Vida e do Ambiente, Universidade de Trás-os-Montes e Alto Douro, Vila Real, Portugal

**Keywords:** fish bacterial diseases, host colonization, host-pathogen interaction, photobacteriosis, trimeric autotransporter adhesins, virulence factors

## Abstract

**Background:**

*Photobacterium damselae* subsp. *piscicida* (*Phdp*) causes severe septicemic infections in marine fish and substantial economic losses in aquaculture. However, the bacterial factors required for host colonization remain poorly characterized.

**Methods:**

Comparative SDS-PAGE protein profiling of *Phdp* field isolates followed by mass spectrometry led to the identification of candidate trimeric autotransporter adhesins (TAAs), which were subsequently characterized using molecular, functional and comparative genomic approaches.

**Results:**

Two previously unrecognized plasmid-encoded TAAs, PadA and PadB, were identified in *Phdp* field isolates and were shown to be expressed during experimental infection of European sea bass (*Dicentrarchus labrax*). Functional assays showed that PadA and PadB increased the resistance of bacterial pellets to disaggregation and bound fish mucus and extracellular matrix components but did not significantly affect biofilm formation. Expression of PadA or PadB in a PadA- and PadB-negative strain did not increase virulence in sea bass. Comparative genomic analysis further identified a third putative TAA, designated PadC, encoded by chromosomal genes in both *P. damselae* subspecies.

**Conclusions:**

By providing the first characterization of TAAs in *Phdp*, this work expands our understanding of surface-associated factors potentially involved in host-pathogen interactions, and establishes a foundation for future studies on the role of these adhesins in photobacteriosis.

## 1 Introduction

*Photobacterium damselae* subsp. *piscicida* (*Phdp*), causes severe bacterial infections in numerous marine fish species and is responsible for substantial economic losses in aquaculture worldwide (Andreoni and Magnani, 2014). Although several virulence-associated factors have been identified in *Phdp*, including the apoptosis-inducing toxin AIP56 (do Vale et al., 2005; Lisboa et al., 2023), iron acquisition systems (Osorio et al., 2010) and secretion systems (do Vale et al., 2017; Abushattal et al., 2022), the molecular mechanisms involved in host colonization and virulence remain poorly understood.

Adhesion is a critical step during bacterial infection, enabling pathogens to establish stable interactions with host surfaces and access favorable niches for persistence and proliferation (Klemm and Schembri, 2000; Pizarro-Cerda and Cossart, 2006). In Gram-negative bacteria, these functions are frequently mediated by surface-exposed adhesins that recognize host cell receptors, mucus components or extracellular matrix (ECM) molecules. However, despite evidence supporting the ability of *Phdp* to interact with host tissues (Magarinos et al., 1996; Remuzgo-Martinez et al., 2014), the bacterial factors mediating these interactions remain poorly characterized.

Trimeric autotransporter adhesins (TAAs) are a family of surface-exposed proteins widely distributed among Gram-negative pathogens and represent some of the best-characterized bacterial adhesins (Linke et al., 2006; Lyskowski et al., 2011; Kiessling et al., 2020). TAAs belong to the type V secretion system (T5SS) and assemble as homotrimeric fibrillar structures composed of a conserved C-terminal membrane anchor, an elongated coiled-coil stalk and a variable N-terminal head domain involved in ligand recognition (Leo et al., 2012; Fan et al., 2016; Meuskens et al., 2019). Classical members of this family contribute to host colonization and virulence, such as *Yersinia* adhesin A (YadA), *Bartonella* adhesin A (BadA), *Neisseria* adhesin A (NadA), *Neisseria* hia/hsf homologue (NhhA), *Haemophilus influenzae* adhesin (Hia) and *Acinetobacter* trimeric autotransporter adhesin (Ata) (El Tahir and Skurnik, 2001; Comanducci et al., 2002; Schmid et al., 2004; Capecchi et al., 2005; Scarselli et al., 2006; Schutz et al., 2010; Muller et al., 2011; Bentancor et al., 2012; Mikula et al., 2012; Chauhan et al., 2016; Atack et al., 2020).

A hallmark of TAAs is their multifunctionality. These proteins frequently mediate adhesion to host cells and ECM components such as collagen, laminin and vitronectin, facilitating bacterial attachment and tissue colonization (Tahir et al., 2000; Scarselli et al., 2006; Leo et al., 2010; Schutz et al., 2010; Muller et al., 2011; Bentancor et al., 2012; Singh et al., 2014; Atack et al., 2020; Meuskens et al., 2021). In addition, several TAAs promote bacterial aggregation, microcolony formation, biofilm development, immune evasion and persistence within the host (Eitel and Dersch, 2002; Scarselli et al., 2006; Sjolinder et al., 2008; Bentancor et al., 2012; Trunk et al., 2018; Okaro et al., 2019; Weidensdorfer et al., 2019). Owing to their surface exposure and central role in host-pathogen interactions, TAAs are widely recognized as important virulence determinants and attractive targets for vaccine development (Comanducci et al., 2002; Winter and Barenkamp, 2010; Weidensdorfer et al., 2019; Sun et al., 2022; Abitbol et al., 2024).

Despite the importance of TAAs in many Gram-negative pathogens, their occurrence and biological function in *P. damselae* subsp. *piscicida* and subsp. *damselae* have remained largely unexplored. Here, we describe two previously uncharacterized proteins from *Phdp* field isolates that display characteristic features of TAAs. These proteins, designated PadA and PadB (*Photobacterium* adhesins A and B, respectively), are encoded by genes carried on plasmids and are absent from some laboratory-maintained strains. Bioinformatic analysis revealed that both proteins comprise the structural hallmarks of typical TAAs, including YadA-like anchor domains and extended repetitive regions, suggesting a potential role in host-pathogen interactions. We also show that PadA and PadB are expressed during infection, increase the resistance of bacterial pellets to disaggregation and interact with several ECM proteins. Finally, using comparative genomic analysis we identified PadC, a novel chromosome-encoded putative TAA, in both *Photobacterium damselae* subspecies.

## 2 Materials and methods

### 2.1 Bacterial strains and culture conditions

*Photobacterium damselae* subsp. *piscicida* (*Phdp*) strains were stored at -80 °C in Tryptic Soy Broth (TSB, Labkem) supplemented with NaCl to a final concentration of 1% (w/v) (TSB-1) and 30% (v/v) glycerol. Recovery from frozen stocks was performed by culturing at 25 °C on Tryptic Soy Agar (TSA, Labkem) containing 1% (w/v) NaCl (TSA-1), or TSA-1 supplemented with 5 µg/mL chloramphenicol (Cm, Calbiochem). *Listeria monocytogenes* (wild type strain EGD-e; (Pinheiro et al., 2018)) was cultured at 25 °C on TSA-1 plates or in TSB-1.

### 2.2 Mass spectrometry

Protein bands were excised from a Coomassie Blue-stained SDS-polyacrylamide gel containing total extracts from *Phdp* GRDL19-1 (Freitas et al., 2022), recovered from an outbreak affecting European sea bass (*Dicentrarchus labrax*) in Greece in 2019. Excised bands were reduced with dithiothreitol (DTT), alkylated with iodoacetamide as previously described (Gomes et al., 2013), and digested with trypsin. The resulting peptides were desalted and concentrated using reverse phase C18 tips (ZipTips, Millipore) following the manufacturer’s instructions, eluted in 60% acetonitrile (ACN)/0.1% (v/v) trifluoroacetic acid, and allowed to dry (SpeedVac, Thermo Scientific, Waltham, MA, USA). The resolubilized peptides were analyzed by liquid chromatography (LC) coupled to an Orbitrap Q-Exactive mass spectrometer (Thermo Scientific) using a nano spray ionization source (Easy-Spray, Thermo Scientific). Reverse phase peptide separation was performed with an Ultimate 3000 system (Thermo Scientific) fitted with a trapping cartridge (Acclaim PepMap C18 100 Å, 5 mm × 300 μm i.d., 160454, Thermo Scientific) in a mobile phase of 2% ACN, 0.1% (v/v) formic acid at 10 µL/min following a separation approach as previously described (Teixeira et al., 2023). The raw data were processed using Proteome Discoverer software (Thermo Scientific) and searched against UniProt database for the taxonomic selection of *Photobacterium* (November 2019 release). The Sequest HT search engine was used to identify tryptic peptides. The ion mass tolerance was 10 ppm for precursor ions and 0.02 Da for fragment ions. Up to two missing cleavage sites were allowed. Cysteine carbamidomethylation was set as a constant modification. Methionine oxidation and N-terminal protein acetylation were defined as variable modifications.

### 2.3 Detection of *padA* and *padB* in *Phdp* field isolates

PCR detection of *padA* and *padB* was evaluated in 50 isolates recovered during *Phdp* outbreaks that occurred in Europe between 2014 and 2020 (**Supplementary Table S1**). Strain MT1415, isolated from European sea bass in Italy (do Vale et al., 2003) was used as a negative control. Bacterial stocks were plated on TSA-1 plates and grown for 48 h at 25 °C. A small portion of bacterial mass was removed with a sterile swab and resuspended in 250 µL distilled water. PCR was performed using NZYTaq II 2x Green Master Mix (NZYTech), with primers pairs *padA*_fwd/*padA*_rev or *padB*_fwd/*padB*_rev (**Supplementary Table S2**) and 0.5 µL of the bacterial suspension as template, with the following program: (a) denaturation for 3 min at 95 °C, (b) 35 cycles of denaturation at 94 °C for 30 s, annealing at 56 °C for 30 s, elongation at 72 °C for 60 and 90 s for *padB* and *padA*, respectively and (c) a final elongation step at 72 °C for 10 min. PCR products were analyzed by electrophoresis in a 1% (w/v) agarose gel. The conservation of the *padA*- and *padB*-specific primer-binding regions was assessed by nucleotide sequence alignments using the available *padA* and *padB* sequences from European, Asia-Pacific and USA *Phdp* genomes (**Supplementary Figure S1**).

### 2.4 Cloning and production of recombinant PadA and PadB proteins

Recombinant PadA and PadB protein variants produced in this study are shown in **Supplementary Figure S2** and the primers used for generating the corresponding expression plasmids are listed in **Supplementary Table S2**. To obtain constructs for expressing full-length PadA (PadA^M1-Q454^) and PadB (PadB^M1-W382^), the respective coding sequences were amplified from total DNA of *Phdp* GRDL19–1 and cloned into the NcoI/XhoI restriction sites of pET28a (Novagen), in frame with a C-terminal 6xHis-tag, yielding plasmids pET28_PadA^M1-Q454^ and pET28_PadB^M1-W382^. The coding regions of PadA^E22-V368^, PadA^V228-V368^, PadB^A28-H305^, PadB^A28-A206^ and PadB^V172-A327^ were PCR-amplified using pET28_PadA^M1-Q454^ or pET28_PadB^M1-W382^ as templates and cloned into the same vector in frame with a C-terminal 6xHis-tag. The DNA sequence encoding PadA^A109-E266^ was codon-optimized for heterologous expression in *Escherichia coli*, synthesized commercially (GenScript Corporation) and cloned into NcoI/XhoI restriction sites of pET28a in frame with a C-terminal 6xHis-tag.

PadA^E22-V368^, PadA^A109-E266^ and PadB^A28-A206^ were expressed in *E. coli* BL21-CodonPlus(DE3)-RIL (Stratagene), whereas PadA^V228-V368^ was produced in *E. coli* Rosetta(DE3) (Novagen). PadB^A28-H305^ and PadB^V172-A327^ were expressed in *E. coli* BL21(DE3)-Star (Invitrogen). Transformed *E. coli* cells were grown in Lysogeny Broth (LB) with appropriate antibiotics at 37 °C with shaking (180 rpm) using culture volumes of 1 L for PadA^E22-V368^ and PadB^A28-H305^, 2 L for PadB^A28-A206^, 3 L for PadA^A109-E266^ and 6 L for PadA^V228-V368^ and PadB^V172-A327^. Protein expression was induced at an OD_600_ ~ 0.6 by adding IPTG to 0.5 mM, and cultures were incubated for an additional 20 h at either 25 °C (PadA^E22-V368^ and PadB^A28-H305^) or 17 °C (all other variants). Cells were harvested by centrifugation, resuspended in 50 mM Tris-HCl pH 8.0 with 500 mM NaCl (40 mL per liter of bacterial culture), and stored at -20 °C. After thawing, cells were lysed by sonication and centrifuged to obtain soluble and insoluble fractions. PadA^A109-E266^, PadA^V228-V368^, PadB^A28-A206^ and PadB^V172-A327^ were purified from the soluble fraction using nickel‐affinity chromatography (Ni‐NTA agarose, ABT) followed by size-exclusion chromatography (Superose 12 10/300 GL and Superdex200 Increase 10/300 GL, GE Healthcare). Size-exclusion chromatography was used to separate putative oligomeric forms from higher-order aggregates and lower-molecular-weight species, and fractions corresponding to the putative oligomeric forms were pooled and concentrated for subsequent binding assays. Representative SEC profiles are shown in **Supplementary Figure S3**. Recombinant PadA^E22-V368^ and PadB^A28-H305^ used as antigens for antibody production were purified using protein-specific procedures. PadA^E22-V368^ was recovered from inclusion bodies, solubilized in guanidine hydrochloride, purified by Ni-NTA under denaturing conditions and dialyzed against phosphate-buffered saline (PBS), leading to protein precipitation. The precipitated protein was recovered by centrifugation and resuspended in PBS. PadB^A28-H305^ from the soluble fraction was applied to a Ni-NTA column under native conditions but remained firmly bound to the resin despite elution with 400 mM imidazole. Therefore, it was recovered from the resin by incubation for 5 min at 95 °C in gel loading buffer (50 mM Tris-HCl pH 8.8, 2% (w/v) SDS, 10% (v/v) glycerol, 0.017% (w/v) bromophenol blue, 2 mM EDTA pH 8.8, 100 mM DTT). To obtain highly purified antigen preparations, both proteins were further purified by preparative SDS-PAGE, and recovered from the polyacrylamide gel as previously described (do Vale et al., 2005). Protein purity was determined by densitometry analysis of Coomassie Blue-stained SDS-PAGE gels using Image Lab software (BioRad). All protein preparations used in this study were at least 90% pure.

### 2.5 Protein quantification

Concentrations of soluble recombinant proteins were determined using a NanoDrop 1000 and/or NanoDrop One (Thermo Scientific) by measuring absorbance at 205 nm and calculating protein concentrations according to the method of Scopes (Anthis and Clore, 2013). Recombinant PadA^E22-V368^ and PadB^A28-H305^ preparations obtained following preparative SDS-PAGE and protein extraction from the gel were quantified by densitometry. Different amounts of each protein preparation were resolved by SDS-PAGE alongside the GRS Unstained Protein Marker (GRISP Research Solutions). Band intensities were quantified using Image Lab software (Bio-Rad) and protein concentrations were estimated by comparison with the 40-kDa marker band.

### 2.6 Complemented MT1415 strains expressing PadA or PadB

MT1415 strains expressing PadA or PadB were generated using a previously described gene complementation strategy (do Vale et al., 2017; Lisboa et al., 2021). Briefly, DNA fragments encoding PadA or PadB preceded by their respective promoter regions were PCR-amplified using total DNA from *Phdp* GRDL19–1 as template and cloned into the BamHI restriction site of pMRB24. After sequence verification, the resulting plasmids were transformed into *E. coli* S17-1-λpir and mobilized into a rifampicin-resistant derivative of *Phdp* MT1415 (MT1415-Rf^R^; (do Vale et al., 2017)) by conjugation, yielding strains MT1415-PadA and MT1415-PadB. An MT1415-Rf^R^ strain carrying the empty vector pMRB24 (MT1415-Empty) was generated as a control. Primers used for cloning and sequencing are listed in **Supplementary Table S2**. To obtain growth curves, bacteria were cultured on TSA-1 plates (MT1415-Rf^R^) or TSA-1 plates containing 5 µg/mL Cm (MT1415-PadA and MT1415-PadB) for 48 h at 25 °C, suspended in TSB-1 at an OD_600_ = 0.5, and inoculated 1:100 in TSB-1 (MT1415-Rf^R^) or TSB-1 containing 5 µg/mL Cm (MT1415-PadA and MT1415-PadB). Aliquots from each culture were transferred, in duplicate, to wells of a 24-well flat bottom plate subsequently covered with a Breath-Easy membrane and the OD_600_ was measured at 1-h interval over 41 h using a Synergy 2 Plate Reader (Biotek) operating at 25 °C with continuous slow shaking. Bacterial growth kinetics were analyzed by fitting OD_600_ measurements obtained over time to the Modified Gompertz model (Zwietering et al., 1990) using non-linear regression. The maximum specific growth rate (*µ*_max_) was estimated from the mean of two technical replicates for each of three independent biological experiments, and the resulting *µ*_max_ was used for statistical analysis.

### 2.7 Protease protection assays

*Phdp* MT1415-Rf^R^, MT1415-PadA and MT1415-PadB were grown on TSA-1 plates for 48 h at 25 °C and resuspended in 20 mM Tris pH 8.0, 300 mM NaCl at OD_600_ = 0.9-1.0. These suspensions were centrifuged at 5,000 *g* for 5 min at room temperature (RT) and the pellets resuspended in 1/5 of the initial volume of the same buffer. Aliquots of these suspensions were left untreated or treated with 10 µg/mL proteinase K (PK, MB13503, NZYTech) for 10 min or 30 min at RT, either as intact cells or after permeabilization for 15 min at 4 °C with 1% (v/v) Triton X-100 (T8787, Sigma). PK was inactivated with 500 µg/mL phenylmethylsulphonyl fluoride (PMSF, P7626, Sigma). Samples were mixed with SDS-PAGE loading buffer, incubated at 98 °C for 15 min and subjected to SDS-PAGE.

### 2.8 Generation of antibodies against PadA and PadB proteins

Rabbit polyclonal antibodies against PadA and PadB were produced by ProteoGenix (France) using purified recombinant PadA^E22-V368^ and PadB^A28-H305^ as antigens. For each antigen, rabbits received four subcutaneous immunizations at two-week intervals, each containing 0.25 mg of recombinant protein. Antigens were administered in PBS with complete Freund’s adjuvant for the first immunization and incomplete Freund’s adjuvant for the three subsequent boosts. Final sera were collected on day 51 and subjected to affinity purification using Protein A. Specificity of the resulting antibodies (Rabbit anti-PadA, Lot N° 15723-121721-A02, 1.52 mg/mL and Rabbit anti-PadB, Lot N° 15723-121721-A03, 2.38 mg/mL) was assessed by western blotting using the recombinant antigens and lysates from MT1415-Rf^R^, MT1415-PadA and MT1415-PadB (**Supplementary Figure S4**).

### 2.9 Fish

European sea bass with no previous history of *Phdp* infection were used in this study. The fish were purchased from commercial hatcheries and maintained in 600 L seawater aquaria at a temperature of 20 ± 1 °C, salinity of 23-28‰, and a photoperiod of 14 h light:10 h dark. Water quality was maintained with mechanical and biological filtration and ozone-disinfection. The fish were fed on commercial pellets (Skretting), according to the supplier’s recommendations. During at least 15 days after receiving the fish, their health status was monitored by observing external appearance, swimming behavior, and appetite. The intra-peritoneal (i.p.) injection of bacterial inocula was performed under anesthesia achieved by immersion in 0.03% (v/v) 2-phenoxyethanol, and euthanasia was performed by immersion in 0.06% (v/v) 2-phenoxyethanol, followed by exsanguination. All experimental protocols involving fish were carried out in accordance with European and Portuguese legislation for the use of animals for scientific purposes (Directive 2010/63/EU; Decreto-Lei 113/2013) and were licensed by the DGAV (Lic. 0421/000/000/2021).

### 2.10 Experimental infections

To prepare the inocula for infections, bacteria were grown on TSA-1 plates (for MT1415-Rf^R^ and GRDL19-1) or TSA-1 plates containing 5 µg/mL Cm (all other strains) for 48 h at 25 °C, resuspended in TSB-1 to an OD_600_ of 0.5-0.6, inoculated 1:100 in TSB-1, grown overnight at 25 °C, 160 rpm, centrifuged, resuspended in TSB-1 and adjusted to the desired bacterial concentrations. Injected doses were confirmed by plating serial dilutions of the inocula on TSA-1 plates. Each fish was injected with 100 µL of bacterial suspension in TSB-1. To determine the contribution of PadA and PadB to virulence, two independent infection experiments were performed. In the first experiment, groups of 10 sea bass (17.4 ± 4.0 g) were i.p. injected with 4.0 x 10^6^ colony-forming units (CFU) of MT1415-Empty, 3.7 x 10^6^ CFU of MT1415-PadA or 2.1 x 10^6^ CFU of MT1415-PadB. In the second experiment, groups of 40 sea bass (96.6 ± 16.9 g) were i.p. injected with 2.2 x 10^7^ CFU of MT1415-Rf^R^, 2.2 x 10^7^ CFU of MT1415-PadA or 1.9 x 10^7^ CFU of MT1415-PadB. Fish were monitored for 10–15 days and mortalities were recorded. Fish showing signs of advanced disease (darkening of body color, lethargy, erratic swimming, and loss of equilibrium) were euthanized and counted as dead. To investigate PadA and PadB expression during infection, sea bass were i.p. injected with 5.4 x 10^6^, 5.4 x 10^5^ or 7.0 x 10^4^ CFU of the field isolate GRDL19-1. Head kidneys and spleens were collected from fish euthanized after reaching advanced disease, between 2–5 days post-infection and processed for western blotting.

### 2.11 Aggregation and disaggregation assays

Strains MT1415-Empty, MT1415-PadA and MT1415-PadB were grown on TSA-1 plates containing 5 µg/mL Cm for 48 h at 25 °C, resuspended in TSB-1 to an OD_600_ of 0.5-0.6 and inoculated 1:100 in TSB-1. Cultures were grown at 25 °C, 160 rpm, until late exponential phase (OD_600_ 0.9-1.0). For the static aggregation assays, 10 mL of cultures were transferred to 15 mL tubes, vortexed for 15 s and then left to stand undisturbed. At 0, 1, 2 and 4 h, 100 µL aliquots were removed from the top of the suspensions, in duplicate, and transferred to a 96-well plate that was maintained on ice. The OD_600_ of the samples was measured using either a Synergy 2 Plate Reader (Biotek) or Synergy Mx Multi-Mode Reader (Biotek). For the disaggregation assays, cultures obtained as described above were diluted in TSB-1 to obtain an OD_600_ of 0.5. Five milliliter aliquots were then centrifuged in 15 mL tubes at 3,220 *g*, 5 min at 4 °C. Tubes were then incubated on a Stuart SRT6 roller mixer operating at 33 rpm at RT. To monitor disaggregation, at 0, 30, 45, 60, 90 and 120 min, triplicate aliquots were collected for OD_600_ measurement, as described for the aggregation assays.

### 2.12 Biofilm formation assay

Biofilm formation was determined using the crystal violet-based assay as previously described (Vivas et al., 2008; Remuzgo-Martinez et al., 2014), with some modifications. The assay was performed using Nunclon Delta surface-treated 24-well polystyrene plates (Thermo Scientific). Bacterial suspensions with an OD_600_ of 0.5, prepared as described in section 2.10 were diluted 1:10 in TSB-1. Five hundred µL of these suspensions were transferred to the plate wells containing 500 µL TSB-1. Wells with sterile TSB-1 were used as negative controls. Plates were incubated at 25 °C in static conditions. After 3 or 6 days, the supernatant was carefully removed, and the wells washed twice with 1 mL distilled water (dH2O). After air-drying, attached bacteria were stained for 30 min with 250 µL/well of 0.5% (w/v) crystal violet (Merck). The staining solution was removed, the wells washed thrice with 1 mL dH2O and the crystal violet solubilized with 250 µL of 96% (v/v) ethanol. Aliquots were transferred to a 96-well plate, and the optical densities at 595 nm measured in a Synergy 2 Plate Reader (Biotek) or Synergy Mx Multi-Mode Reader (Biotek).

### 2.13 Preparation of sea bass mucus

Skin and intestinal mucus were collected post-mortem from six European sea bass fasted for 24 h before sampling. Skin mucus was collected by gently scraping the body surface with a cell scraper. For intestinal mucus collection, the intestine was removed, opened longitudinally, and the luminal surface was gently scraped with a cell scraper. Skin and intestinal mucus samples were pooled separately, diluted in sea bass PBS (sbPBS; PBS supplemented with 42 mM NaCl) and centrifuged (3,184 *g*, 15 min, 4 °C) to remove scales and debris. Supernatants were aliquoted, frozen in liquid nitrogen, and stored at -80 °C. Protein concentrations were determined using a BCA Protein Assay Kit (Thermo Scientific), following the manufacturer’s instructions for the microplate procedure and using BSA as the standard.

### 2.14 ELISA

Collagen IV (human), gelatin (porcine), laminin (mouse) and casein (bovine) were purchased from Sigma-Aldrich (C7521, G2500, L2020 and C8654, respectively). Collagen I (rat tail), vitronectin (human) and BSA (Fraction V) were purchased from Corning (354236), Thermo Scientific (AF-140-09) and Biochemika (MB04602), respectively. Flat-bottom 96-well MaxiSorp plates (Thermo Scientific) were coated overnight at 4 °C with 50 µL of sea bass skin or intestinal mucus (1 mg/mL total protein in PBS), ECM proteins (collagen I and IV, laminin and vitronectin) or gelatin (10 µg/mL in PBS). BSA and casein were included as non-ECM reference proteins. Following washing with PBS containing 0.05% (v/v) Tween 20 (PBS-T), wells were incubated with the indicated PadA or PadB protein variants (5 μM) for 1 h at RT, 100 rpm. After washing 5 times with PBS-T, wells were incubated for 1 h at RT with 50 µL rabbit anti-PadA (1:1 000) or anti-PadB (1:2 000) antibodies diluted in PBS-T containing 1% (w/v) BSA. Subsequently, wells were washed and incubated for 1 h at RT (100 rpm) with HRP-conjugated sheep anti-rabbit IgG (The Binding Site, AP311; 1:2 000). After washing, 50 µL TMB/E Ultra Sensitive, Blue, Horseradish Peroxidase Substrate (Millipore) were added, and the reaction was stopped by adding 50 µL of 0.3 M H_2_SO_4_. Absorbance was measured at 450 and 570 nm using a Synergy 2 Plate Reader (Biotek) or Synergy Mx Multi-Mode Reader (Biotek). Final absorbance values were obtained by subtracting the absorbance at 570 nm from that at 450 nm. Wells coated as described above but receiving vehicle instead of PadA or PadB protein variants were used as blanks. Wells coated with the PadA or PadB protein variants were included as technical positive controls to verify antibody detection.

### 2.15 SDS-PAGE and western blotting

SDS-PAGE was performed using the Laemmli discontinuous buffer system (Laemmli, 1970) as previously described (do Vale et al., 2005). For analysis of whole-cell protein profiles, aliquots of bacterial cultures were collected by centrifugation (5,000 *g*, 5 min, 4 °C), the supernatants discarded, and the bacterial pellets stored at -20 °C until analysis. Pellets were resuspended in gel loading buffer (50 mM Tris-HCl pH 8.8, 2% (w/v) SDS, 10% (v/v) glycerol, 0.017% (w/v) bromophenol blue, 2 mM EDTA pH 8.8, 100 mM DTT), incubated for 5 min at 95 °C and subjected to electrophoresis in 8, 12 or 14% denaturing polyacrylamide gels. Proteins in the gels were stained with Coomassie Brilliant Blue R-250 and gel images were obtained using a GS-900 Calibrated Densitometer (Bio-Rad) coupled to the Image Lab Software, version 6.1 (Bio-Rad). For characterization of the rabbit anti-PadA and anti-PadB antibodies (**Supplementary Figure S4**), recombinant proteins and whole-cell lysates from MT1415, MT1415-PadA and MT1415-PadB were subjected to SDS-PAGE as described above and transferred to nitrocellulose membranes. Transfer efficiency and protein loading were assessed by Ponceau S staining. Membranes were blocked in Tris-buffered saline (TBS) containing 5% low-fat powdered milk and 0.1% (v/v) Tween 20 (TBS-T) for 30–60 min at RT, incubated with rabbit anti-PadA or anti-PadB antibody diluted 1:20,000 in blocking buffer, and immunoreactive bands detected using an alkaline phosphatase-conjugated anti-rabbit IgG secondary antibody (Sigma-Aldrich, A9919; 1:10,000 in blocking buffer) and BCIP/NBT (S3771, Promega). For detection of PadA and PadB in infected fish tissues, head kidneys and spleens from fish showing signs of advanced disease or from non-infected control fish were homogenized in SDS-PAGE sample buffer (10 µL/mg tissue) using an Ultra-Turrax T25 homogenizer (Janke & Kunkel, Germany). Tissue homogenates were subjected to SDS-PAGE and western blotting as described above, except that immunoreactive bands were detected using HRP-conjugated goat anti-rabbit IgG (Cell Signaling Technology, #7074; 1:2,000 in blocking buffer) and SuperSignal™ West Dura Extended Duration Substrate (Thermo Scientific).

### 2.16 Whole-genome sequencing and assembly

Total DNA was extracted from the field isolates GRDL19-1, ITDL19-1, SPDL19‐3 and SPSA19-2 (**Supplementary Table S1**) grown on TSA-1 plates for 24–48 h at 25 °C using the Maxwell^®^ RSC Blood DNA Kit and the Maxwell^®^ RSC automated extraction system (Promega), according to the manufacturer’s instructions. DNA concentration, purity and integrity were assessed using a Qubit 4 fluorometer (Thermo Scientific), NanoDrop spectrophotometer (Thermo Scientific) and Agilent TapeStation Genomic DNA ScreenTape system, respectively. Sequencing libraries were prepared using the Oxford Nanopore Rapid Barcoding Kit (SQK-RBK114.24), multiplexed on the same R10.4.1 FLO-MIN114 flow cell and sequenced on a MinION device. Basecalling was performed using Dorado (v5.0.0, high-accuracy model). Reads were quality filtered (minimum Q score 15) using Filtlong, adapter-trimmed with Porechop, and reads shorter than 1.5 kb were discarded. Genome assemblies were generated using Autocycler (v0.2.1; Flye v2.9.6-b1802; miniasm v0.3-r179; raven v1.8.3), annotated using the NCBI Prokaryotic Genome Annotation Pipeline (PGAP), and deposited in GenBank under BioProject accession number PRJNA1499273.

### 2.17 *In silico* protein analysis

The predicted amino acid sequences of PadA, PadB and PadC from *Photobacterium damselae* subsp. *piscicida* strain GRDL19-1 (**Supplementary Table S3**) were used for all protein sequence and structural analysis. Protein sequences were analyzed using InterPro (https://www.ebi.ac.uk/interpro/) and HHhrepID (Zimmermann et al., 2018; Gabler et al., 2020). Structural predictions were generated using AlphaFold3 (Abramson et al., 2024). For PadA and PadB, the predicted N-terminal signal peptides were removed prior to structural modeling. Comparative models were built with MODELLER (Sali and Blundell, 1993; Fiser et al., 2000; Marti-Renom et al., 2000; Webb and Sali, 2016) using the YadA structural model YadAmodv2.pdb as template. The model was obtained from the Max Planck Institute for Biology Tübingen structural models repository (https://www.bio.mpg.de/176408/structures).

### 2.18 Comparative genome analysis

PadA-, PadB- and PadC-related sequences were identified by BLASTp searches using the corresponding protein sequences from *Phdp* GRDL19–1 as queries against all *Photobacterium damselae* genomes available in the NCBI RefSeq proteins database (accessed July 2026) together with the four European *Phdp* genomes generated in this study. Amino acid sequence identity and the genomic location of the corresponding coding genes were determined for each related sequence. For comparative analysis of the genomic context, genomic regions extending up to 10 kb upstream and downstream of *padA*, *padB* or *padC* were extracted from the corresponding genome assemblies and compared using EasyFig (Sullivan et al., 2011). When contigs did not span the full region, the available sequence was used.

### 2.19 Statistical analysis

For continuous data, comparisons between two groups were performed using Student’s t-test or Welch’s t-test when the assumption of homogeneity of variances was not met. Comparisons among three or more groups were performed using one-way or two-way analysis of variance (ANOVA) followed by Tukey’s multiple comparisons test. For *in vivo* infection assays, survival proportions were compared using the chi-square test or Fisher´s exact test. The statistical tests applied to each data set are indicated in the corresponding figure legends. Statistical analysis were performed using IBM SPSS Statistics. Differences were considered statistically significant when *p* < 0.05. Graphs were prepared using GraphPad Prism 8.

## 3 Results

### 3.1 Identification of PadA and PadB, two plasmid-encoded trimeric autotransporter adhesins from *Phdp*

Comparative SDS-PAGE profiling of European field isolates of *Photobacterium damselae* subsp. *piscicida* (*Phdp*) revealed the widespread presence of two prominent high-molecular-weight protein bands that were absent in the laboratory-maintained virulent strain MT1415 (**Figure 1A**). These bands were excised from a gel loaded with strain GRDL19–1 and analyzed by mass spectrometry, identifying two previously uncharacterized proteins with predicted molecular masses of approximately 54 and 41 kDa. Sequence analysis revealed that both proteins contain a C-terminal domain homologous to the anchor domain of *Yersinia* adhesin A (YadA; pfam03895), a hallmark of trimeric autotransporter adhesins (TAAs). In addition, both proteins displayed apparent molecular masses on SDS-PAGE that are much larger than the theoretical values, probably reflecting the existence of SDS-resistant oligomers, a property described for other members of the TAA family. Based on these findings, the proteins were designated PadA (*Photobacterium* adhesin A) and PadB (*Photobacterium* adhesin B).

**Figure 1:**
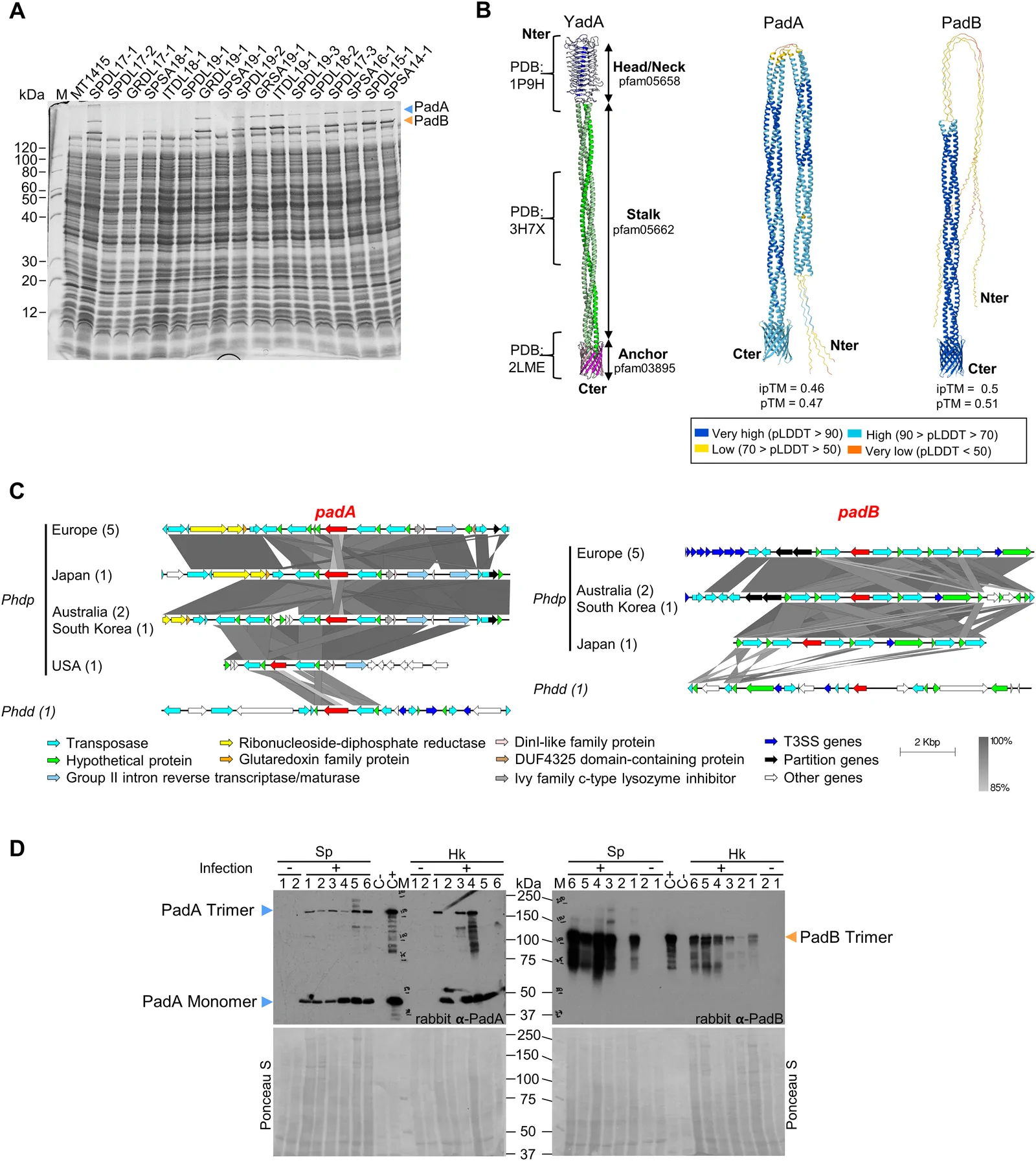
Identification, structural models, genomic context and *in vivo* expression of PadA and PadB in *Photobacterium damselae* subsp. *piscicida* (*Phdp*). **(A)** Identification of PadA and PadB in European field isolates of *Phdp*. SDS-PAGE profiles of whole-cell extracts from European field isolates (origin and year of isolation are indicated in **Supplementary Table S1**) and the laboratory-maintained strain MT1415. Each lane was loaded with whole-cell extract equivalent to 150 μL of bacterial culture, prepared in SDS-containing gel loading buffer and heated for 5 min at 95 °C before electrophoresis. The gel was stained with Coomassie Blue. Bands corresponding to PadA and PadB are indicated by blue and orange arrowheads, respectively. Numbers on the left indicate the molecular weight of the markers (M), in kDa. **(B)** AlphaFold3 predicted structures of PadA and PadB. Left, cartoon representation of the structural model of the trimeric autotransporter adhesin YadA (YadAmodv2.pdb) based on experimentally resolved partial structures (PDB 1P9H, 3H7X and 2LME), showing the head/neck (cyan, pfam05658), the stalk (green, pfam05662) and the membrane anchor (magenta, pfam03895). Predicted trimeric structures of PadA (middle) and PadB (right). The models are colored according to the predicted local distance difference test (pLDDT) score: blue, very high confidence (pLDDT > 90); cyan, high confidence (70 < pLDDT ≤ 90); yellow, low confidence (50 < pLDDT ≤ 70); and orange, very low confidence (pLDDT ≤ 50). The N- and C-termini are indicated. Predicted template modelling (pTM) and interface predicted template modelling (ipTM) scores are shown below each model. AlphaFold3 structure predictions were generated using the PadA and PadB protein sequences from *Phdp* strain GRDL19–1 after removal of the predicted N-terminal signal peptides (PadA^E22-Q454^ and PadB^A28-W382^). **(C)** Comparison of the genomic context surrounding the *padA* and *padB* loci among the *Phdp* genomes sequenced in this study and publicly available *Photobacterium damselae* genomes carrying *padA*- and/or *padB-*related sequences. Strains sharing the same genomic organization were grouped into a single schematic for clarity. The number of the strains with each organization are indicated in parentheses, and the corresponding strains are listed in **Supplementary Table S4**. Genes are shown as arrows indicating the direction of transcription and are colored according to their predicted functions. Grey-shaded blocks connect homologous regions, and color intensity indicating nucleotide sequence identity (85%-100%). The positions of *padA* and *padB* are highlighted in red. Pairwise BLASTn comparisons were visualized using EasyFig. **(D)** Detection of PadA and PadB in spleen and head-kidney of *Phdp*-infected sea bass by western blotting. Total lysates from spleen (Sp) and head-kidney (Hk) of six infected and two non-infected control fish were analyzed using specific anti-PadA or anti-PadB antibodies. Lysates from MT1415-Empty (C-) and MT1415-PadA or MT1415-PadB (C+) were included as negative and positive controls, respectively. Ponceau S staining is shown as a loading control. Blue and orange arrowheads indicate PadA and PadB, respectively.

To gain insight into the structural organization of PadA and PadB, AlphaFold3 structure predictions were generated using the corresponding protein sequences from *Phdp* strain GRDL19–1 after removal of the predicted N-terminal signal peptides. The predicted structures showed high-confidence C-terminal β-barrel-like membrane anchor and elongated coiled-coil stalk regions, consistent with the overall architecture of trimeric autotransporter adhesins (**Figure 1B**). In contrast, the N-terminal regions were predicted with low confidence and appeared largely disordered. Comparative modeling with MODELLER generated trimeric assemblies comprising a head domain, an extended coiled-coil stalk and a C-terminal membrane anchor for both PadA and PadB (**Supplementary Figure S5**) (Nummelin et al., 2004; Koretke et al., 2006). However, the use of the YadA structure as a template in MODELLER may have imposed a YadA-like architecture on PadA and PadB. Therefore, experimental structural studies will be required to establish the native architecture of PadA and PadB, particularly the organization of their N-terminal regions. Sequence analysis also identified eight highly conserved tandem repeat units in PadA and a collagen triple-helix repeat region (pfam01391) containing the characteristic glycine-rich G-X-Y motif of bacterial collagen-like domains in PadB (**Supplementary Figure S6**).

BLASTp searches using PadA and PadB from strain GRDL19–1 as queries identified related sequences in multiple publicly available *Phdp* genomes and in the four European *Phdp* genomes sequenced in this study (**Supplementary Table S4**). In *Phdd*, related sequences were identified in three genome assemblies corresponding to the same type strain (ATCC 33539 = CIP 102761 = NCTC 11647) (Sapio et al., 2026). PadA-related sequences identified in *Phdp* were highly conserved, displaying 95.8-100% amino acid identity. PadA-related sequences identified in the *Phdd* type strain showed 91.0-93.1% amino acid identity with GRDL19–1 PadA. The PadB-related sequences identified in *Phdp* were also highly conserved (99-100% amino acid identity), whereas the PadB-related sequences identified in the three genome assemblies representing the *Phdd* type strain were more divergent, showing approximately 60% amino acid identity with the GRDL19–1 PadB protein (**Supplementary Table S4**). Among the *Phdp* genomes yielding at least one PadA- or PadB-related BLASTp hit, most harbored both sequences. OT-51443 and 91–197 strains harbored only PadA-related sequences, whereas PP3 only a PadB-related sequence. MT1415 lacked both. The adhesin genes were located on plasmids in all fully assembled genomes analyzed. In all available complete European *Phdp* genomes, *padA* and *padB* were located on plasmids of ~160–170 kb (**Supplementary Table S4**) that share extensive sequence similarity with the previously described plasmid pPHDPT3 from the Japanese strain PP3 (Abushattal et al., 2022). In the *Phdd* type strain (CIP 102761 assembly), the PadA- and PadB-related sequences were located on the 203-kb plasmid pPHDD203 (**Supplementary Table S4**) (Vences et al., 2017). Comparative genomic analysis of the regions surrounding *padA* and *padB* revealed that both genes are located within highly dynamic plasmid-associated regions containing multiple transposase genes (**Figure 1C**). The close association with mobile genetic elements suggests that these loci may be prone to genomic rearrangements that may facilitate horizontal gene transfer, likely contributing to the acquisition, diversification, or loss of adhesin-associated regions. Among strains harboring *padA* or *padB*, the corresponding loci were highly conserved across geographically distinct *Phdp* field isolates from Europe, Australia, South Korea, Japan and the USA. In contrast, the corresponding adhesin loci identified in the *Phdd* type strain displayed a distinct genomic organization relative to *Phdp* (**Figure 1C**). PCR screening of 50 recent *Phdp* field isolates recovered from natural outbreaks in different fish species and geographical locations (**Supplementary Table S1**) detected *padA* and *padB* in 36% and 42% of isolates, respectively. Nucleotide sequence comparison showed that the primer-binding regions were identical across the available European, Asia-Pacific and USA *Phdp padA* and *padB* sequences (**Supplementary Figure S1**). Nevertheless, because genome sequence information is not available for all isolates included in the PCR screening, the possibility of false-negative results due to sequence variation in the primer-binding regions cannot be excluded. In addition, since the virulence plasmid pPHDPT3 from *Phdp* strain PP3, which shares extensive sequence similarity with the *padA*- and *padB*-carrying plasmids from other *Phdp* strains, is known to be unstable and prone to recombination and loss during *in vitro* culture (Abushattal et al., 2022; Rudenko et al., 2026), non-detection of *padA* and *padB* in some cultured field isolates may reflect plasmid loss during laboratory propagation. Thus, the observed PCR detection frequencies may underestimate the distribution of these genes in natural populations.

To determine whether PadA and PadB are expressed during infection, spleen and head-kidney lysates from sea bass experimentally infected with the hypervirulent *Phdp* field isolate GRDL19–1 were analyzed by western blotting using specific anti-PadA and anti-PadB antibodies. Immunoreactive bands corresponding to both proteins were detected in tissues from infected fish but were absent in non-infected controls (**Figure 1D**), confirming *in vivo* expression of both adhesins during *Phdp* infection.

### 3.2 Expression of PadA or PadB does not increase virulence in the *Phdp* MT1415 background

Attempts to generate stable isogenic mutants lacking *padA* and/or *padB* were unsuccessful because the plasmid encoding these TAAs exhibited marked instability during *in vitro* cultivation, consistent with previous reports for the closely related virulence plasmid pPHDPT3 (Abushattal et al., 2022). Therefore, to investigate the potential biological role of these adhesins, an alternative gain-of-function strategy was adopted by complementing the PadA- and PadB-negative strain MT1415-Rf^R^ with plasmids encoding *padA* or *padB*.

SDS-PAGE analysis confirmed the expression of PadA or PadB in the corresponding complemented derivatives, with both proteins migrating predominantly as high-molecular-weight SDS-resistant species, consistent with the oligomeric assembly characteristic of surface-exposed trimeric autotransporter adhesins (**Figure 2A**). The surface exposure of the recombinant adhesins was further confirmed by protease protection assays. For this purpose, suspensions containing intact bacteria or bacteria pre-treated with Triton X-100 (a detergent that permeabilizes lipid bilayers) were treated with proteinase K (PK) and analyzed by SDS-PAGE. In both MT1415-PadA and MT1415-PadB, the high-molecular-weight adhesin species were susceptible to PK treatment in the absence of Triton X-100, indicating that the proteins are correctly exported and assembled at the bacterial surface (**Figure 2B**). Of note, while PadB was completely degraded, PadA showed only a slight shift following PK treatment. However, the same pattern was observed after membrane permeabilization with Triton X-100, indicating that the partial digestion was not due to impaired accessibility of protease K to PadA but rather to intrinsic resistance of this protein to the protease. Note the markedly different protein profiles observed after Triton X-100 pretreatment, confirming that intracellular proteins were protected from PK digestion in intact cells. Apart from the presence of the expected adhesin-associated bands, the overall protein profiles of the complemented strains were similar to those of the parental MT1415 strain (**Figure 2A**). In addition, growth was comparable among all strains (**Figure 2C**).

**Figure 2:**
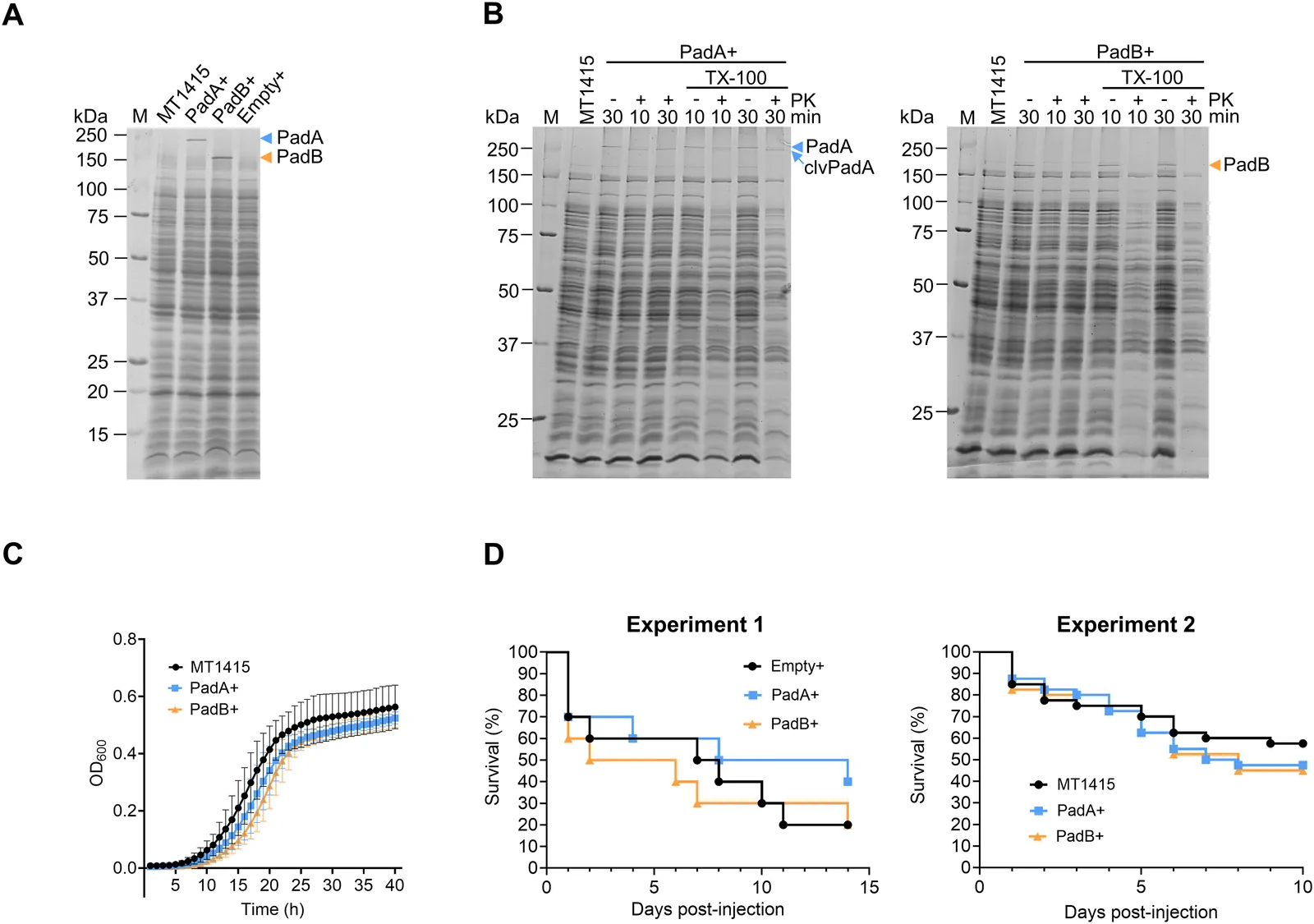
Expression of PadA or PadB by *Phdp* MT1415 does not result in increased virulence for sea bass. **(A)** SDS-PAGE analysis of whole-cell extracts from the parental strain MT1415, and MT1415 transformed with pMRB24 encoding *padA* (PadA+), *padB* (PadB+) or the empty vector (Empty+). Each lane was loaded with total bacterial extracts equivalent to 150 μL of bacterial culture prepared in SDS-containing gel loading buffer and heated for 5 min at 95 °C before electrophoresis. The gel was stained with Coomassie Blue. PadA and PadB bands are indicated by blue and orange arrowheads, respectively. Numbers on the left indicate the molecular weight of the markers (M), in kDa. **(B)** Protease protection assay of MT1415, PadA+ and PadB+ strains. Whole cells were left untreated or incubated with 10 µg/mL proteinase K (PK) for 10 or 30 min in the presence or absence of Triton X-100 (TX-100), followed by SDS-PAGE analysis. Each lane was loaded with sample equivalent to 150 μL of bacterial culture prepared in SDS-containing gel loading buffer and heated for 15 min at 98 °C before electrophoresis. The gel was stained with Coomassie Blue. Numbers on the left indicate the molecular weight of the markers (M), in kDa. PadA and PadB bands are indicated by blue and orange arrowheads, respectively and cleaved PadA (clvPadA) is indicated by blue arrow. **(C)** Growth curves of the indicated strains grown at 25 °C in TSB-1 (MT1415) or TSB-1 containing 5 μg/mL chloramphenicol (PadA+, PadB+). Data represent the mean ± SD of three independent experiments performed with technical duplicates. No statistically significant differences in maximum specific growth rate (µmax) were detected among the strains (ANOVA, *p* > 0.05). **(D)** Kaplan-Meier survival curves of sea bass intraperitoneally infected with the indicated strains in two independent experiments. Experiment 1: Empty+ (4.0 x 10^6^ CFUs/fish), PadA+ (3.7 x 10^6^ CFUs/fish) and PadB+ (2.1 x 10^6^ CFUs/fish); fish weight, 17.4 ± 4.0 g, (n = 10/group). Experiment 2: MT1415 (2.2 x 10^7^ CFUs/fish), PadA+ (2.2 x 10^7^ CFUs/fish) and PadB+ (1.9 x 10^7^ CFUs/fish); fish weight, 96.6 ± 16.9 g, (n = 40/group). Within each experiment, no significant differences in survival proportions were observed among the groups (chi-square test, *p* > 0.05).

The contribution of PadA and PadB to virulence was evaluated in two independent sea bass infection experiments using intraperitoneal inoculation. In both trials, no significant differences in mortality among fish infected with MT1415-PadA, MT1415-PadB or MT1415-Empty were observed (chi-square test, *p >* 0.05), indicating that expression of either adhesin was insufficient to enhance virulence in the MT1415 background under the tested conditions (**Figure 2D**). Although these results suggest that PadA and PadB are not *per se* key virulence factors, the contribution of these adhesins to virulence during natural infection or in the context of fully virulent *Phdp* strains cannot be excluded. Indeed, the MT1415-derived strains used in these experiments lack the ~160-170-kb plasmid carrying *padA* and *padB*, which shares extensive sequence similarity with the previously described pPHDPT3 plasmid, reported to encode several genes associated with *Phdp* pathogenicity, including a type III secretion system essential for full virulence in *Phdp* (Abushattal et al., 2022). The importance of coordinating TAA-mediated adhesion with other virulence mechanisms is well illustrated in *Yersinia*, where efficient type III secretion requires compatibility between the length of the YadA adhesin and the T3SS injectisome needle (Mota et al., 2005). In addition, the intraperitoneal inoculation protocol used in these assays bypasses the initial host colonization and mucosal adhesion steps that occur in natural infections, where TAAs are likely to play a more relevant role.

### 3.3 PadA and PadB increase bacterial pellet resistance to disaggregation and bind ECM components

Since several TAAs have been associated with bacterial aggregation, cell-to-cell adhesion and biofilm formation (Linke et al., 2006; Kiessling et al., 2020), we investigated whether PadA and PadB contribute to these phenotypes in *Phdp*. Static aggregation assays performed with MT1415 derivatives expressing PadA or PadB showed no significant differences relative to the MT1415-Empty control strain (**Supplementary Figure S7**), indicating that expression of either adhesin does not promote aggregation of suspended *Phdp* cells under the tested *in vitro* conditions. However, during these experiments, pellets formed by MT1415-PadA and MT1415-PadB were consistently more difficult to resuspend than those of the control strain, suggesting stronger or more stable associations between bacterial cells. To quantitatively evaluate this observation, a pellet disaggregation assay was developed (see Materials and Methods). As shown in **Figures 3A, B**, pellets formed by MT1415-PadA and MT1415-PadB showed significantly greater resistance to dissociation than the MT1415-Empty control, indicating that expression of either adhesin increases the resistance of bacterial pellets to disaggregation under these experimental conditions. Whether this feature plays a role in host colonization and persistence remains to be investigated.

**Figure 3:**
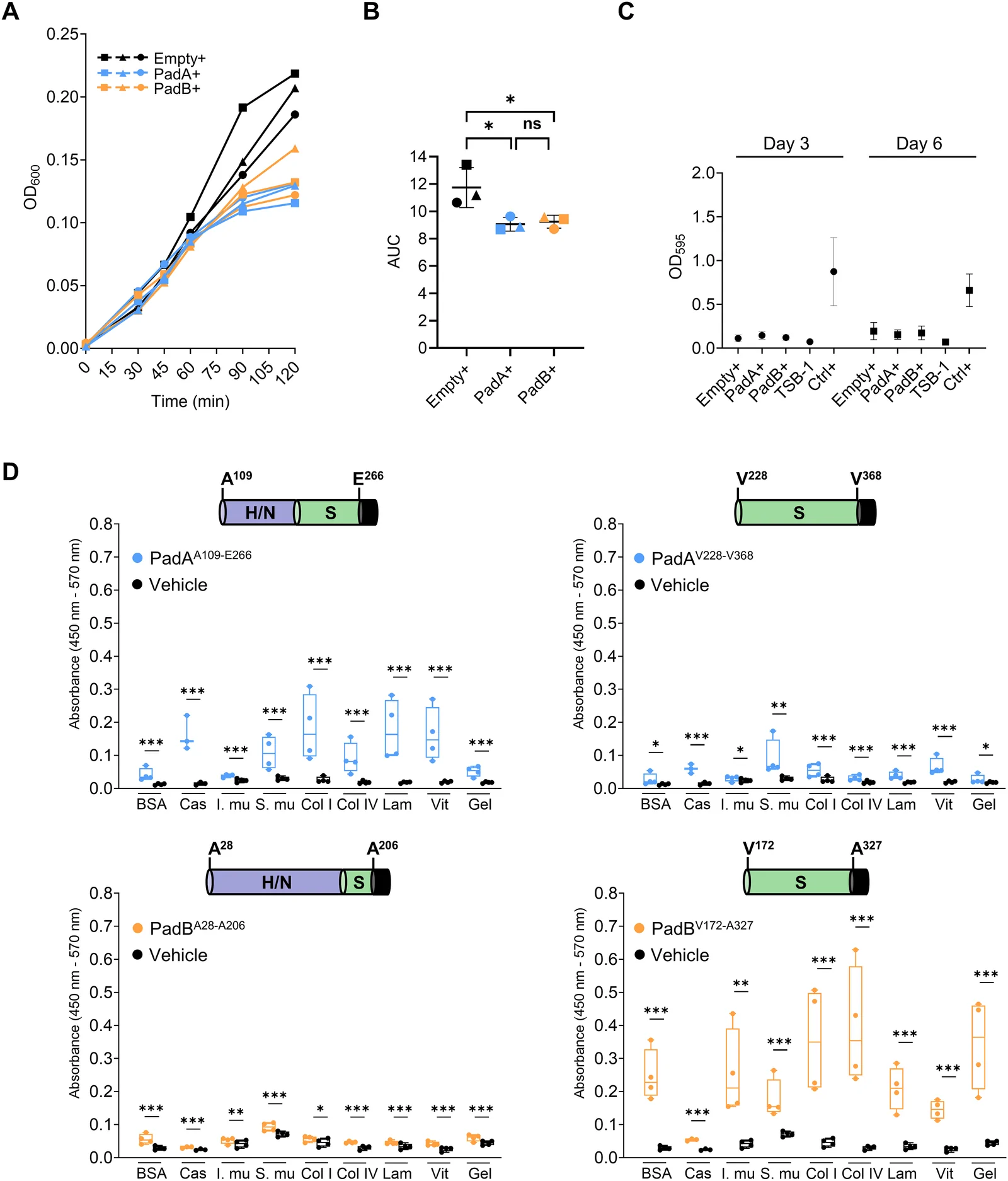
Functional characterization of PadA and PadB: bacterial pellet disaggregation, biofilm formation and binding to extracellular matrix proteins. **(A)** Pellet disaggregation assay of MT1415-derived strains expressing PadA (PadA+), PadB (PadB+) or carrying the empty vector (Empty+). Cultures were pelleted and incubated on a tube roller at room temperature. Pellet dissociation was monitored by measuring the OD_600_ of the supernatant over time. Results from three independent experiments, each performed with technical triplicates, are shown. Results obtained in each independent experiment are represented by the same symbol. **(B)** Cumulative pellet resuspension over time, expressed as the area under the curve (AUC). Results are shown as mean ± SD of three independent experiments. Each symbol represents an independent experiment. Statistical analysis was performed using one-way ANOVA followed by Tukey’s multiple comparisons test. *p < 0.05; ns, non-significant. **(C)** Biofilm formation by MT1415-derived strains expressing PadA (PadA+), PadB (PadB+) or carrying the empty vector (Empty+). Biofilm biomass was quantified after 3 or 6 days by crystal violet staining. *Listeria monocytogenes* EGD-e was included as a positive control for biofilm formation (Ctrl+). Bars represent mean ± SD of four independent experiments, each performed with five technical replicates. Statistical analysis was performed among the *Phdp* strains and the negative control (TSB-1) using two-way ANOVA followed by Tukey’s multiple comparisons test. No significant differences were observed. **(D)** Binding of recombinant PadA and PadB variants to sea bass mucus and extracellular matrix (ECM) proteins assessed by ELISA. Microplate wells coated with the indicated ECM proteins (Col I: collagen I; Col IV: collagen IV; Lam: laminin; Vit: vitronectin), intestinal or skin mucus (I.mu or S.mu, respectively), gelatin (Gel) or the non-ECM reference proteins BSA and casein (Cas) were incubated with the indicated PadA or PadB variants. Results are presented as median and interquartile range from four independent experiments, each with three technical replicates per condition. For each coating condition, binding obtained with each recombinant protein variant was compared with the corresponding vehicle using Student’s t-test or Welch’s t-test when the assumption of homogeneity of variances was not met. *p < 0.05; **p < 0.01; ***p < 0.001.

Given that expression of PadA or PadB was associated with increased resistance of bacterial pellets to disaggregation, we next evaluated whether these adhesins could also contribute to biofilm formation. Biofilm formation assays performed using crystal violet staining demonstrated that MT1415-Empty did not form detectable biofilms after either 3 or 6 days of incubation (**Figure 3C**). This is consistent with previous observations that biofilm formation in *Phdp* is highly strain- and condition-dependent (Remuzgo-Martinez et al., 2014). As shown in **Figure 3C**, expression of PadA- or PadB was not sufficient to induce the formation of biofilms under these conditions.

Some bacterial adhesins facilitate colonization through interaction with host mucus or ECM components (Kaiser et al., 2012; Pimenta et al., 2020). We performed ELISA-based binding assays to evaluate the ability of four recombinant versions of PadA and PadB (PadA^A109-E266^, PadA^V228-V368^, PadB^A28-A206^ and PadB^V172-A327^; **Supplementary Figure S2**) to interact with sea bass mucus and selected ECM proteins, including collagen I and IV, laminin, vitronectin and gelatin. Binding profiles differed among the tested PadA and PadB protein variants (**Figure 3D**). PadA^A109-E266^ displayed preferential binding to collagen I, laminin, vitronectin and skin mucus, whereas PadB^V172-A327^ interacted more broadly with both mucus preparations and all ECM proteins tested. In contrast, PadA^V228-V368^ and PadB^A28-A206^ showed weaker binding signals, although statistically significant interactions were detected. Interestingly, PadA^A109-E266^ and PadB^V172-A327^ also differed in their interactions with the non-ECM control proteins, with the former binding preferentially to casein and the latter showing higher binding to BSA (**Figure 3D**). Together, these results highlight differences in substrate binding among the recombinant PadA and PadB variants.

### 3.4 Identification of a novel chromosome-encoded putative trimeric autotransporter adhesin, PadC

To further investigate the diversity of TAAs in *Photobacterium damselae*, BLASTp searches were performed using one of the more divergent *Phdd* sequences identified in the initial PadB-based search (accession number: WP_005306612.1), which shares approximately 60% amino acid identity with PadB from the *Phdp* strain GRDL19-1 (**Supplementary Table S4**). This analysis led to the identification of a previously unrecognized putative TAA, which we designated as PadC. Comparative genomic analysis identified highly conserved *padC*-related sequences in *Phdp* and *Phdd* strains from diverse geographic origins and host species including the *padA*- and *padB*-negative strain MT1415 used in this study (**Supplementary Table S5**). Unlike *padA* and *padB*, which are plasmid encoded, *padC* was consistently located on chromosome 2 in all closed genomes analyzed. In draft assemblies, the gene was identified on unclosed contigs and therefore its exact chromosomal location could not be confirmed. Comparative analysis showed that the genomic context surrounding *padC* is largely conserved among strains (**Figure 4A**).

**Figure 4:**
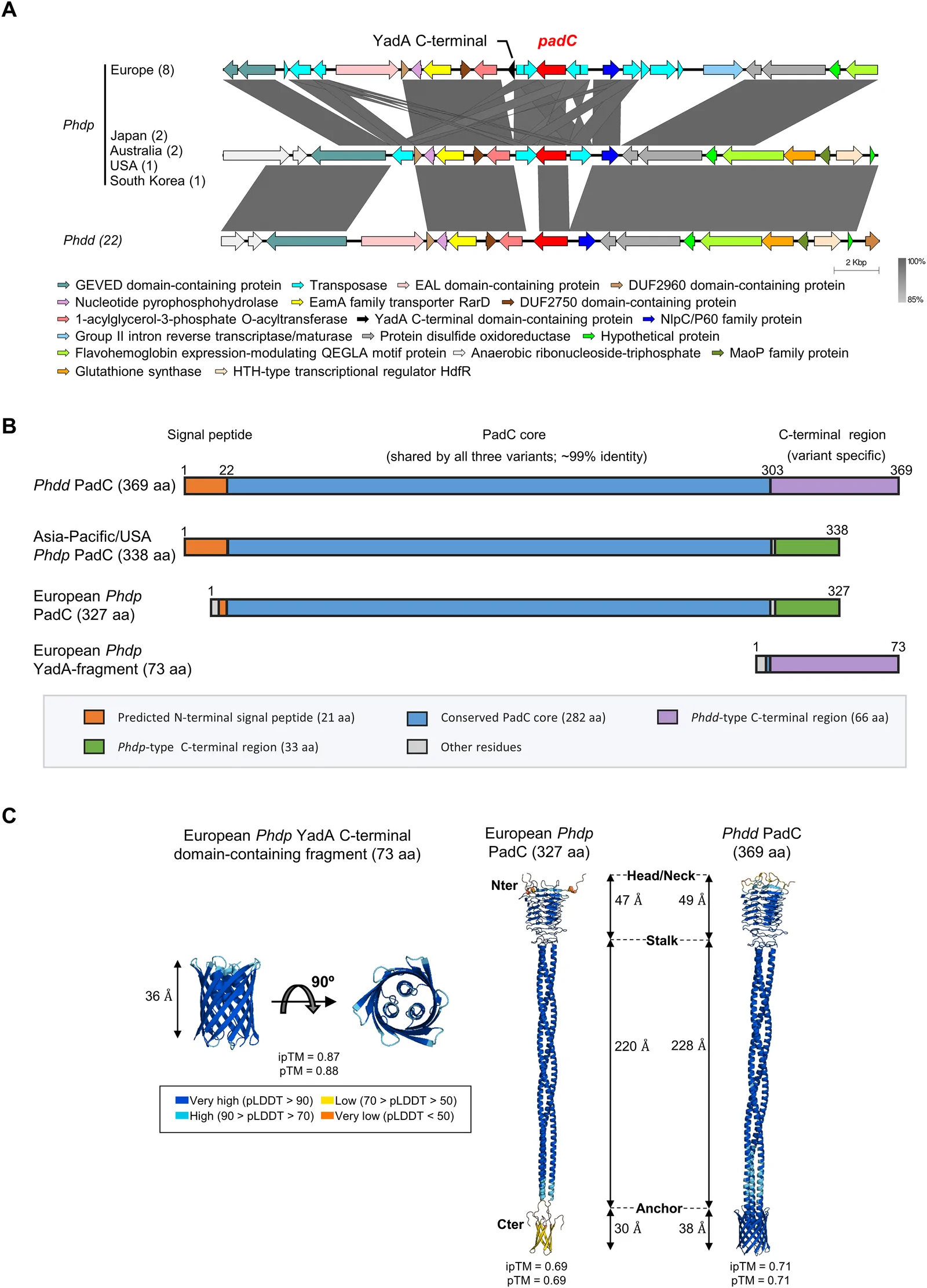
Genomic context and predicted organization of PadC. **(A)** Comparison of the genomic context surrounding the *padC* locus among the *Phdp* genomes sequenced in this study and publicly available *Photobacterium damselae* genomes carrying *padC*-related sequences. Strains sharing the same genomic organization were grouped into a single schematic for clarity. The number of strains represented in each group is indicated in parentheses and the corresponding strains are listed in **Supplementary Table S5**. Genes are shown as arrows indicating the direction of transcription and are colored according to their predicted functions. The *padC* genes are highlighted in red. Gray-shaded blocks connect homologous regions, with the color intensity indicating nucleotide sequence identity (85-100%). Pairwise BLASTn comparisons were visualized using EasyFig. **(B)** Schematic comparison of the predicted amino acid sequences encoded by the different *padC* ORFs shown in **(A)** and by the YadA C-terminal domain-containing ORF identified in European *Phdp* strains. Conserved and variant-specific regions are indicated according to the color key, with boundaries and total sequence lengths shown. In the European *Phdp* PadC sequence, the short orange box immediately preceding the conserved core represents four residues identical to the C-terminal residues of the predicted signal peptide in the Asia-Pacific/USA *Phdp* and *Phdd* sequences, although no signal peptide is predicted for the European sequence. The C-terminal 68 residues of the 73-aa YadA-family fragment are identical to the corresponding terminal region of *Phdd* PadC, spanning the last two residues of the conserved core and the complete 66-aa *Phdd*-type C-terminal region. aa, amino acids. **(C)** AlphaFold3-predicted structures of a YadA C-terminal domain-containing protein from European *Phdp*, European *Phdp* PadC (GRDL19-1; 327 aa) and *Phdd* PadC (WP_237030430.1; 369 aa). For structural modelling, the full-length European *Phdp* PadC sequence was used, while the predicted signal peptide was removed from the *Phdd* PadC sequence. The mature Asia-Pacific/USA *Phdp* PadC sequence (WP_086959024.1) yielded an essentially identical structure over the region shared with the European *Phdp* PadC and is therefore not shown. The YadA-family fragment is shown in side and top views. The models are colored according to the predicted local distance difference test (pLDDT) score: blue, very high confidence (pLDDT > 90); cyan, high confidence (70 < pLDDT ≤ 90); yellow, low confidence (50 < pLDDT ≤ 70); and orange, very low confidence (pLDDT ≤ 50). The N- and C-termini are indicated. Predicted template modelling (pTM) and interface predicted template modelling (ipTM) scores are shown below each model.

Closer examination of the *padC*-related sequences revealed three distinct configurations (**Supplementary Table S6**). European *Phdp* strains contained an identical *padC* ORF predicted to encode a 327-aa protein lacking an N-terminal signal peptide, whereas Asia-Pacific and USA *Phdp* strains carried a distinct *padC* ORF predicted to encode a 338-aa protein with an N-terminal signal peptide. The *Phdd padC* ORFs were predicted to encode a longer, 369-aa protein that also contained an N-terminal signal peptide and a distinct extended C-terminal region (**Figure 4B**). The three predicted amino acid sequences shared a highly conserved 282-aa core, while the two *Phdp* forms displayed a similar C-terminal configuration that differed from the extended C-terminal region of the *Phdd* sequence (**Figure 4B**). Inspection of the corresponding genomic regions did not reveal additional upstream or downstream coding sequence that could account for extension of the shorter *Phdp padC* ORFs. Notably, examination of the underlying nucleotide sequences showed that the shared *Phdp*-type C-terminal region is encoded by sequence corresponding to the terminal region of an ISPda1 insertion sequence.

Inspection of the surrounding genomic context further showed that, despite the overall conservation of local synteny, the *Phdp padC* loci contained several insertions of mobile-element-associated sequences (**Figure 4A**). In the European *Phdp* strains, a separate 73-aa ORF annotated as a YadA C-terminal domain-containing protein was located downstream of *padC*, immediately after a transposase and upstream of the conserved downstream gene cluster. The C-terminal 68 residues of the predicted product were identical to the corresponding terminal region of the 369-aa *Phdd* PadC sequence, encompassing the last two residues of the conserved PadC core and the complete 66-aa *Phdd*-type C-terminal region (**Figure 4B**). This genomic organization is consistent with local rearrangement and remodeling of the *padC* locus.

To assess the structural organization of these predicted proteins, AlphaFold3 models were generated for the full-length European *Phdp* PadC, the mature Asia-Pacific/USA *Phdp* PadC and the mature *Phdd* PadC sequences. The European *Phdp* and *Phdd* PadC models are shown in **Figure 4C**. The mature Asia-Pacific/USA *Phdp* PadC sequence yielded an essentially identical predicted structure, apart from the eight-residue N-terminal extension present in the European *Phdp* model, and is therefore not shown. The predicted models revealed a trimeric autotransporter adhesin architecture composed of a N-terminal head domain, an extended coiled-coil stalk and a C-terminal membrane anchor domain. However, the C-terminal region of the *Phdp* models did not form an unambiguous complete β-barrel-like structure, whereas the *Phdd* PadC model contained a well-defined C-terminal β-barrel predicted with high confidence. In the European *Phdp* form, the additional absence of a predicted N-terminal signal peptide further raises questions about its ability to be exported and assembled as a functional TAA. Notably, the 73-aa YadA C-terminal domain-containing protein identified in the European *Phdp* loci was itself predicted to form a complete β-barrel-like structure (**Figure 4C**).

## 4 Discussion

The present study identifies and characterizes two previously unrecognized trimeric autotransporter adhesins (TAAs), PadA and PadB, in *Photobacterium damselae* subsp. *piscicida* (*Phdp*), and describes a third putative adhesin, PadC, identified in both *P. damselae* subspecies. This represents the first characterization of TAAs in *Phdp*, expanding our understanding of the surface-associated factors potentially involved in host-pathogen interactions in this important fish pathogen.

Several observations strongly support the classification of PadA and PadB as bona fide TAAs. Both proteins contain YadA-like C-terminal anchor domains, display the characteristic domain organization of trimeric autotransporters, and form high-molecular-weight SDS-resistant species consistent with trimeric assembly. Moreover, both adhesins were shown to be surface exposed when expressed in the MT1415 background. Together, these findings provide complementary structural and biochemical evidence that PadA and PadB are authentic TAAs. The presence of multiple repeat motifs in PadA and a collagen-like region in PadB further supports this classification, as similar structural features have been associated with adhesive functions in previously characterized TAAs (Leo et al., 2010; Thibau et al., 2022).

An important finding of this work is that PadA and PadB are expressed *in vivo*, as both proteins were detected in tissues from fish experimentally infected with the *Phdp* strain GRDL19-1. This finding supports a biological role for PadA and PadB during host-pathogen interactions. Notably, despite the dynamic nature of the surrounding plasmid regions, the adhesin coding sequences remained highly conserved among geographically distant *Phdp* isolates, suggesting that selective pressure acts to preserve the functions of PadA and PadB during infection.

TAAs are among the best-characterized adhesins of Gram-negative bacteria and contribute to diverse aspects of pathogenicity, including adhesion to host tissues, autoaggregation, biofilm formation, immune evasion and persistence (Attia et al., 2005; Bullard et al., 2005; Capecchi et al., 2005; Linke et al., 2006; Valle et al., 2008; Lafontaine et al., 2014). However, individual TAAs often perform distinct biological functions depending on the pathogen and host niche. In the present study, expression of PadA or PadB did not increase virulence of the MT1415 strain. Although this result suggests that neither adhesin alone is sufficient to enhance pathogenicity in this experimental model, several experimental limitations should be considered. One of these limitations is the genetic background of the recipient strain, which lacks not only *padA* and *padB*, but the entire ~160–170 kb plasmid carrying these genes in recently isolated European *Phdp* strains. Consequently, the contribution of PadA and PadB may not be fully recapitulated in the MT1415 background. Moreover, intraperitoneal inoculation bypasses the initial stages of infection, including adhesion to mucosal surfaces and tissue colonization, where TAAs are generally expected to play important roles (Klemm and Schembri, 2000; Pizarro-Cerda and Cossart, 2006; Schutz et al., 2010). Consequently, the absence of a virulence phenotype in our assays does not exclude an important role for PadA and PadB during natural infection.

Although PadA and PadB did not promote autoaggregation of suspended bacterial cultures, both adhesins significantly increased resistance of bacterial pellets to dissociation. Similar cell-cell interaction phenotypes have been described for several TAAs, including YadA, BadA and members of the Vomp family, which have been associated with bacterial aggregation, microcolony formation and close interbacterial interactions (Zhang et al., 2004; Schutz et al., 2010; Trunk et al., 2018; Okaro et al., 2019). The increased resistance to pellet disaggregation observed here may therefore reflect stronger or more stable associations between bacterial cells expressing PadA or PadB. This observation is particularly interesting considering that photobacteriosis is characterized by the presence of bacterial aggregates within infected tissues and necrotic lesions (Romalde, 2002), raising the possibility that these adhesins contribute to formation and/or stability of bacterial aggregates during infection.

We also found that expression of PadA or PadB was insufficient to promote biofilm formation under the conditions tested. While several TAAs, including YadA, BadA, Hia and Ata, have been implicated in biofilm development (Bentancor et al., 2012; Okaro et al., 2019; Atack et al., 2020), biofilm formation is a complex phenotype influenced by environmental conditions and the coordinated activity of multiple bacterial factors (Lopez et al., 2010; Trunk et al., 2018). Furthermore, previous studies have shown that biofilm formation in *Phdp* is strain-dependent and not universally observed (Remuzgo-Martinez et al., 2014). The absence of a detectable biofilm phenotype therefore suggests that PadA and PadB alone are insufficient to promote biofilm formation in the MT1415 background, rather than excluding a contribution under alternative conditions.

The binding assays performed with recombinant protein variants revealed statistically significant interactions with fish mucus and ECM components. Such interactions are consistent with one of the best-established functions of TAAs, namely adhesion to host ECM molecules including collagen, laminin and vitronectin (Tahir et al., 2000; Scarselli et al., 2006; Leo et al., 2010; Schutz et al., 2010; Muller et al., 2011; Bentancor et al., 2012; Singh et al., 2014; Atack et al., 2020; Meuskens et al., 2021; Thibau et al., 2022). Interestingly, PadA and PadB displayed different binding preferences, suggesting that these adhesins may recognize distinct host targets. Although the observed interactions were generally modest and some binding towards non-ECM proteins was detected, the overall binding profiles support the ability of PadA and PadB to interact with host extracellular components. Nevertheless, recombinant TAA fragments may not fully reproduce the conformation or binding properties of the corresponding full-length adhesins displayed on the bacterial surface. Future studies using intact bacteria and full-length proteins will therefore be important to validate these interactions and determine their relevance to host colonization.

A particularly noteworthy outcome of this study was the identification of PadC, a previously unrecognized chromosomally encoded putative TAA identified in both *Phdp* and *Phdd* strains. In contrast to the plasmid-encoded adhesins PadA and PadB, PadC is maintained within a largely conserved chromosomal locus. However, comparative sequence and structural analysis revealed distinct PadC configurations across the two subspecies. The *Phdd* PadC sequence contains a predicted N-terminal signal peptide and a well-defined C-terminal β-barrel-like membrane anchor, whereas the *Phdp* forms display an atypical C-terminal architecture that does not form an unambiguous complete β-barrel in the AlphaFold3 models. In addition, the European *Phdp* form lacks a predicted N-terminal signal peptide. Notably, the *Phdp*-type C-terminal region is encoded by sequence corresponding to the terminal region of an ISPda1 insertion sequence, directly linking the atypical C-terminal configuration to the remodeling of this locus by mobile genetic elements. In the European *Phdp* strains, this altered configuration is accompanied by a separate 73-aa YadA C-terminal domain-containing ORF, whose C-terminal 68 residues are identical to the corresponding terminal region of *Phdd* PadC and whose predicted structure forms a complete β-barrel-like domain. Taken together, these features further support the possibility that *padC* has undergone functional inactivation or pseudogenization in *Phdp*. Such a scenario would be consistent with the broader pattern of genome evolution described for *Phdp*, in which extensive insertion sequence proliferation has been associated with chromosomal rearrangements, widespread pseudogenization and gene decay (Balado et al., 2017; Baseggio et al., 2021, 2022).

Overall, the present findings reveal a previously unrecognized repertoire of TAAs in *P. damselae* and provide the first characterization of PadA and PadB in *Phdp*. Future studies employing stable mutants in virulent field isolates and infection models that preserve natural routes of host colonization will be essential to fully determine the contribution of PadA and PadB to the pathogenesis of photobacteriosis. Additionally, it would be worthwhile to evaluate whether these proteins could represent suitable vaccine targets against *Phdp* infections.

## Data Availability

The datasets presented in this study can be found in online repositories. The names of the repository/repositories and accession number(s) can be found below: https://www.ncbi.nlm.nih.gov/genbank/, PRJNA1499273.
