# Supplementary material for: Identification and characterization of trimeric autotransporter adhesins from Photobacterium damselae subsp. piscicida
Source: Front Cell Infect Microbiol. 2026 Oct 30;16:1964857. doi: 10.3389/fcimb.2026.1964857 (PMC13759277; doi:10.3389/fcimb.2026.1964857)
Supplement: Supplementary file 1 [file DataSheet1.pdf]

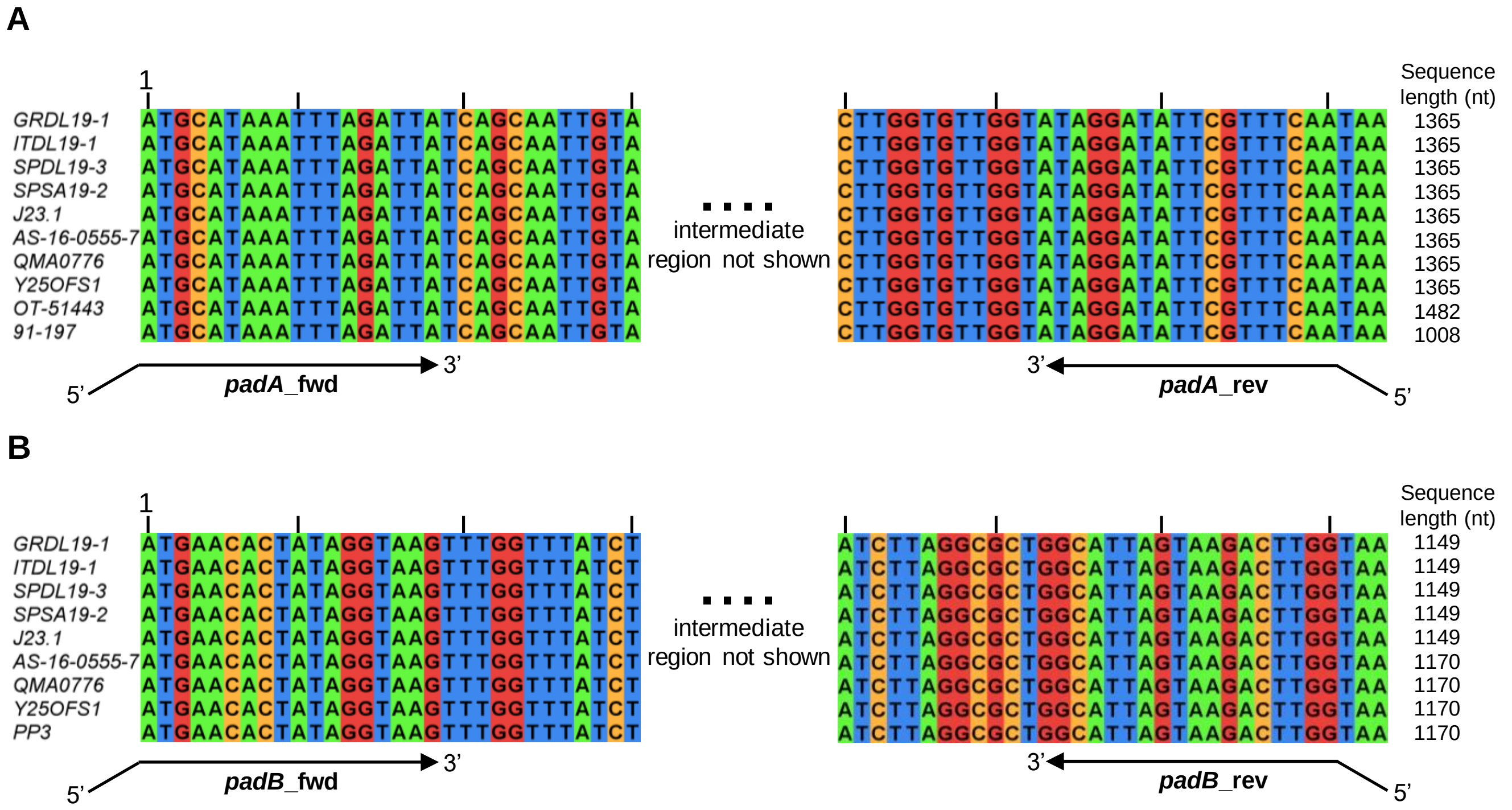

Supplementary Figure S1. Nucleotide sequence conservation of the *padA* and *padB* primer-binding regions among European, Asia-Pacific and USA *Photobacterium damselae* subsp. *piscicida* strains. Multiple sequence alignment of the primer-binding regions of *padA* **(A)** and *padB* **(B)** are shown for European, Asia-Pacific and USA *Phdp* strains for which the corresponding nucleotide sequences were available. The left and right alignment blocks correspond to the 5′ and 3′ ends of the sequences, respectively; the intermediate regions are omitted for clarity. Sequence lengths are indicated on the right. The alignment was performed using Clustal Omega program. Nucleotides are colored according to the Jalview nucleotide coloring scheme, and arrows indicate the orientation of the respective primers used in the PCR screening of *Phdp* field isolates.

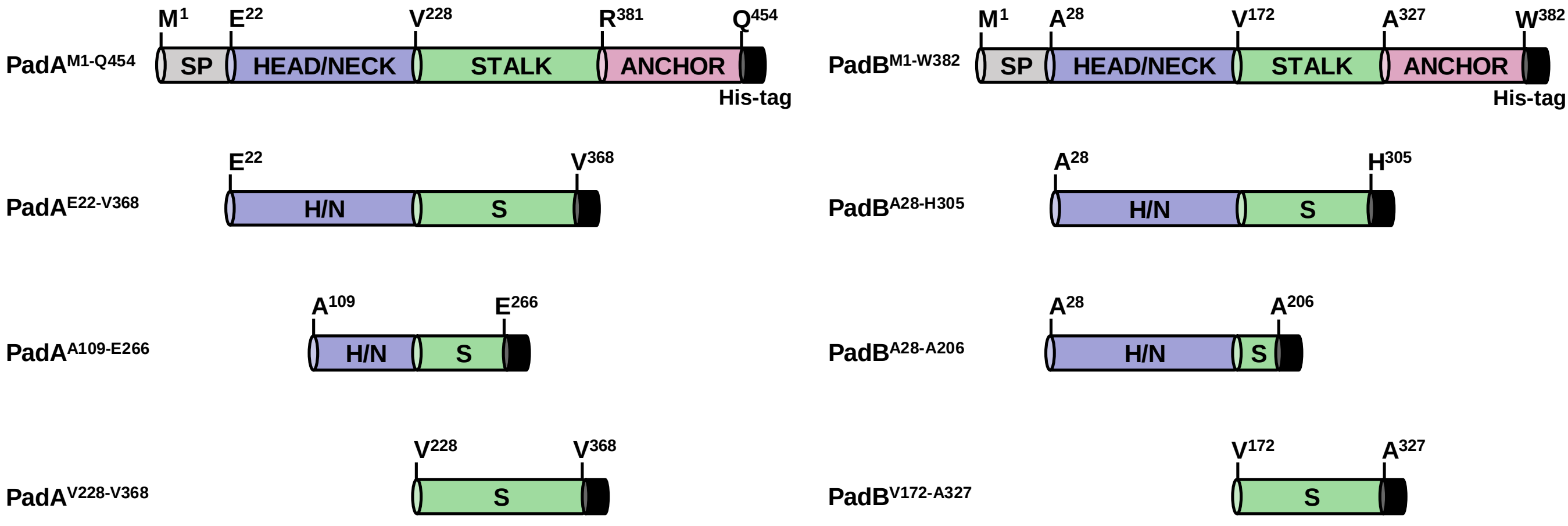

Supplementary Figure S2. Linear representation of recombinant PadA and PadB protein variants used in this study. The full-length variants PadA$^{M1\text{-}Q454}$ and PadB$^{M1\text{-}W382}$ comprise a signal peptide (SP, grey), head/neck (H/N, cyan), stalk (S, green), membrane anchor (pink), and C-terminal 6x Histidine-tag (His-tag, black). Residue numbers in the full-length variants indicate the predicted domain boundaries. In the truncated recombinant variants, residue numbers indicate the N- and C-terminal residues of the expressed proteins and do not necessarily coincide with predicted domain boundaries.

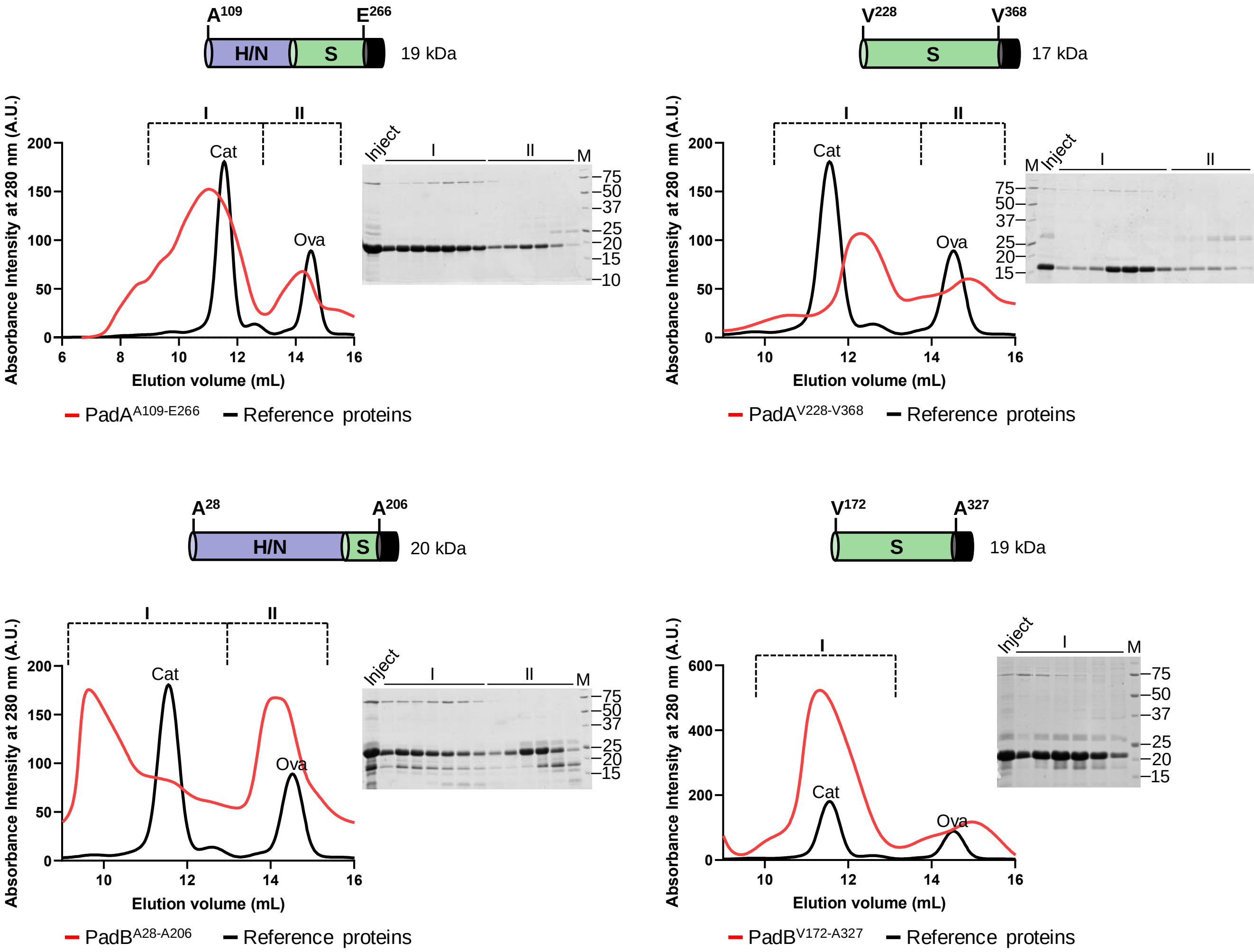

Supplementary Figure S3. Size-exclusion chromatography (SEC) profiles and SDS-PAGE analysis of the indicated recombinant PadA and PadB proteins. Domain organization of the recombinant proteins is depicted above each SEC chromatogram (H/N, head domain; S, stalk domain; black, C-terminal 6×His tag), together with the expected molecular weight of the corresponding monomer. Red traces correspond to the indicated recombinant PadA or PadB proteins, and black traces to the molecular-weight reference proteins catalase (Cat, 240 kDa) and ovalbumin (Ova, 45 kDa). SEC was performed using 50 mM Tris-HCl (pH 8.0) containing 300 mM NaCl as the running buffer. The column void volume (V_0) was 8 mL. Dashed boxes (**I** and **II**) indicate the fractions collected for SDS-PAGE analysis. In each case, fractions within region I correspond to those pooled and concentrated for subsequent binding assays. Gels were stained with Coomassie Brilliant Blue. M, molecular-weight marker. Numbers on the left or right side of the gels indicate molecular mass, in kDa.

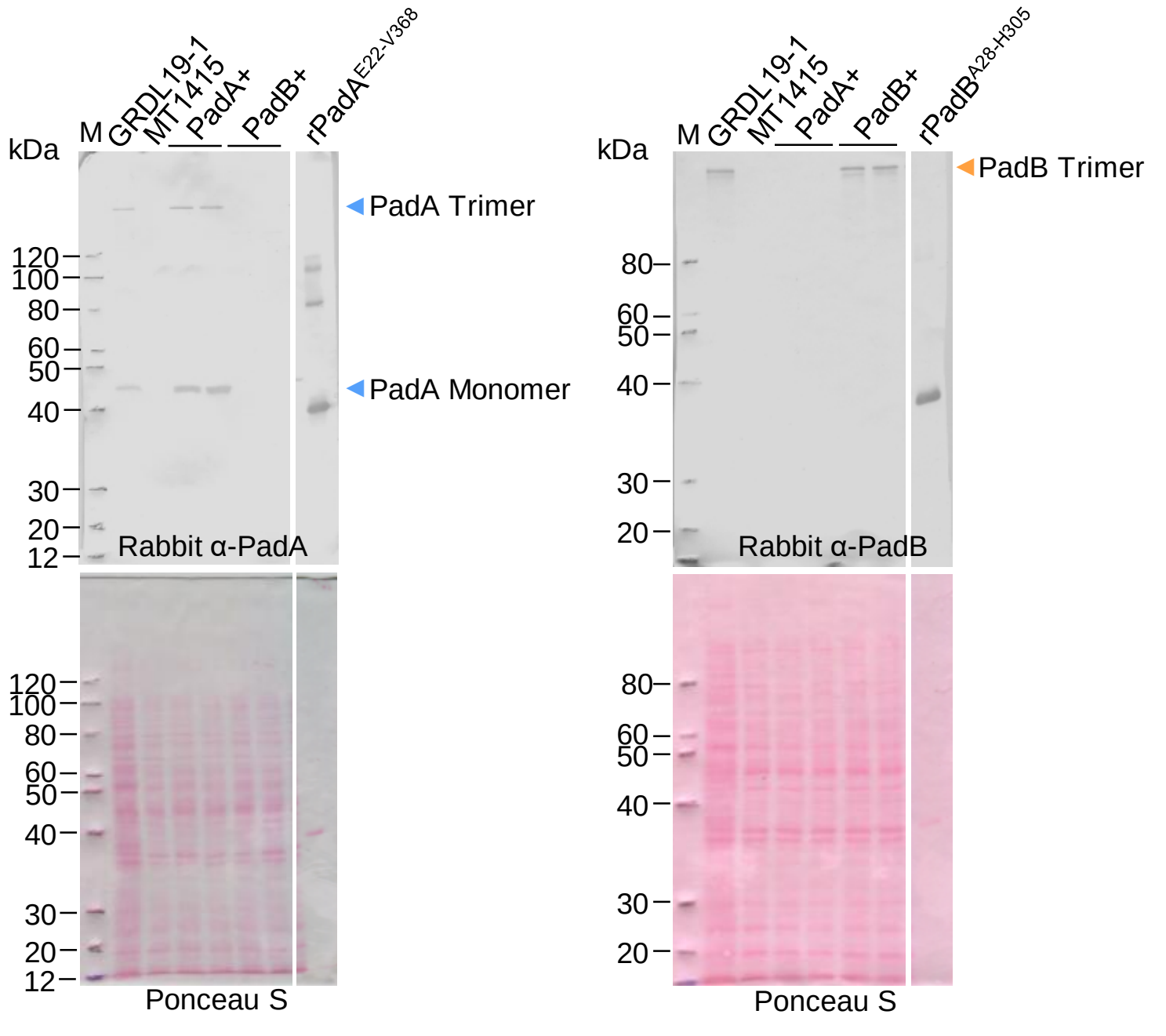

Supplementary Figure S4. Validation of rabbit anti-PadA and anti-PadB polyclonal antibodies by Western blotting. Whole-cell lysates from GRDL19-1, MT1415, two independent MT1415-PadA (PadA+) clones and two independent MT1415-PadB (PadB+) clones, together with purified recombinant $PadA^{E22–V368}$ and $PadB^{A28–H305}$, corresponding to the antigens used for rabbit immunization, were analysed by Western blotting. Membranes were probed with anti-PadA antibody (left) or anti-PadB antibody (right) at 1:20000 dilution. Ponceau S staining is shown below each blot as a loading control. For each antibody, all lanes shown are from the same membrane and were cut and juxtaposed for presentation purposes only. Blue and orange arrowheads indicate PadA and PadB, respectively. Numbers on the left indicate the molecular weight markers (M), in kDa.

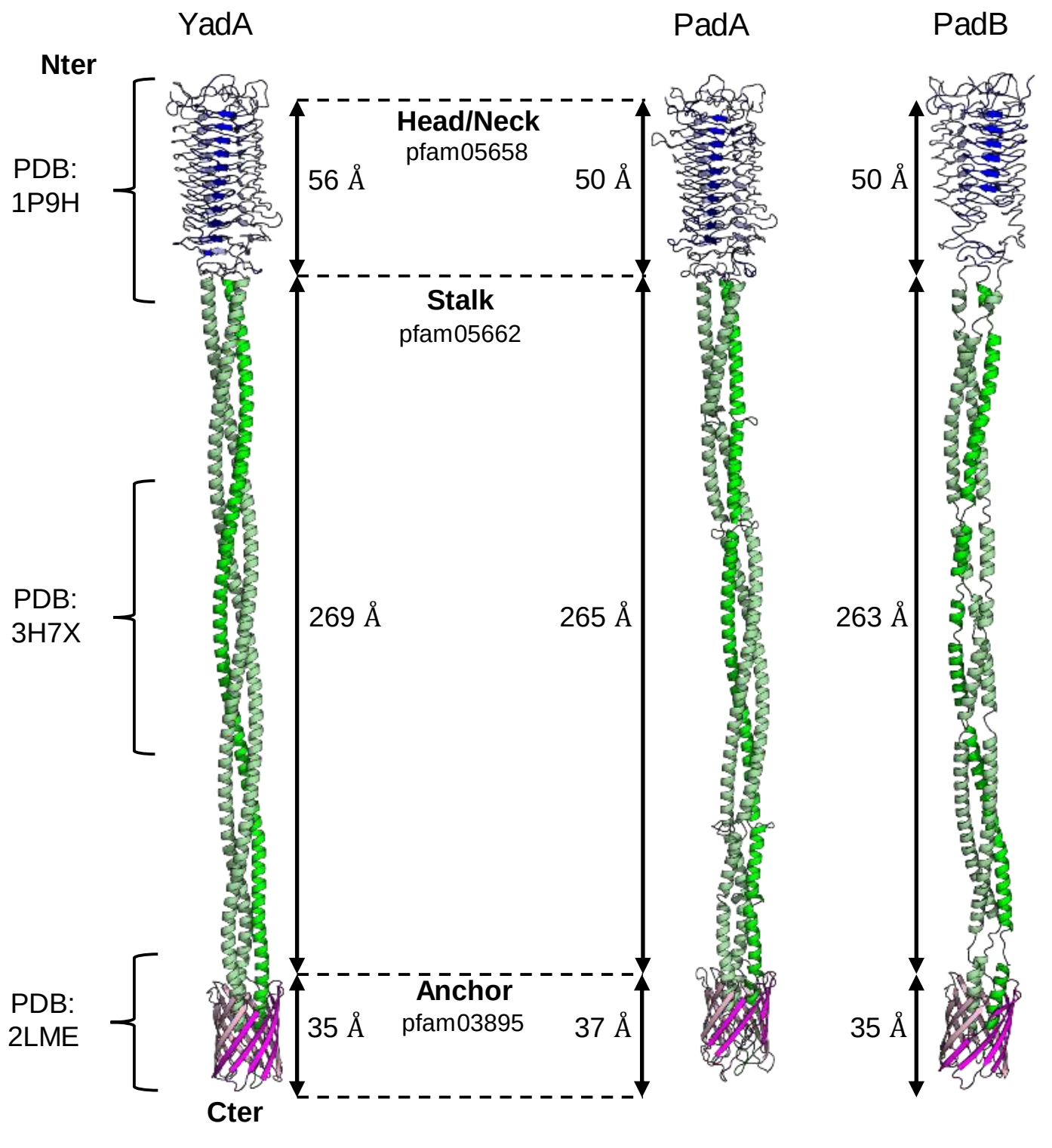

Supplementary Figure S5. MODELLER-based comparative models of PadA and PadB from *P. damselae* subsp. *piscicida* strain GRDL19-1. Left, cartoon representation of the structural model of the trimeric autotransporter adhesin YadA (YadAmodv2.pdb) based on experimentally resolved partial structures (PDB 1P9H, 3H7X and 2LME), showing the head/neck (cyan, pfam05658), the stalk (green, pfam05662) and the membrane anchor (magenta, pfam03895). Right, comparative models of PadA and PadB generated with MODELLER using the YadA structural model as template. The models show the YadA-template-based organization of PadA and PadB into a head domain, an extended coiled-coil stalk and a C-terminal membrane anchor. In each trimer, one monomer is highlighted. The models were built using the predicted mature forms of PadA ($PadA^{E22\text{-}Q454}$) and PadB ($PadB^{A28\text{-}W382}$).

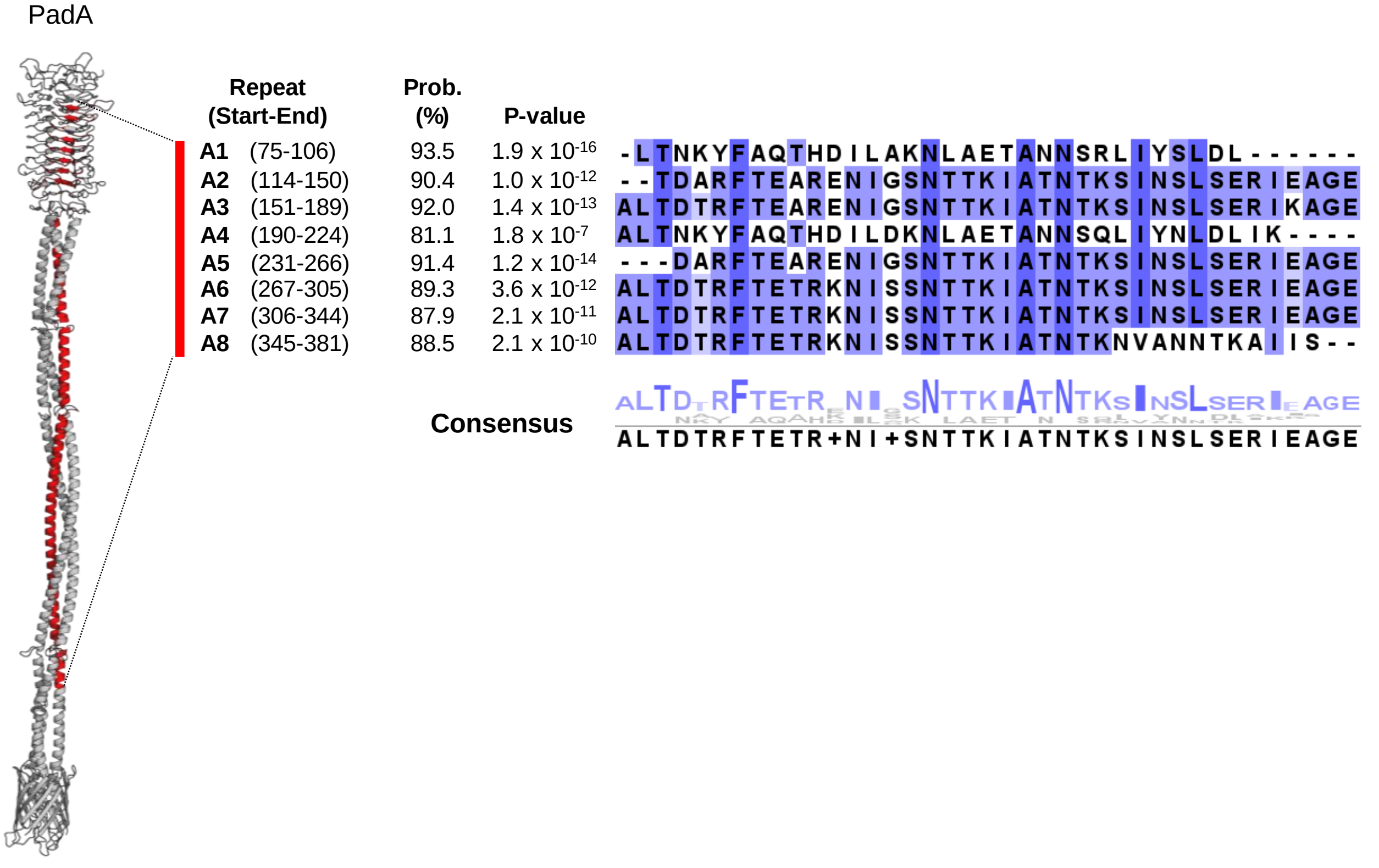

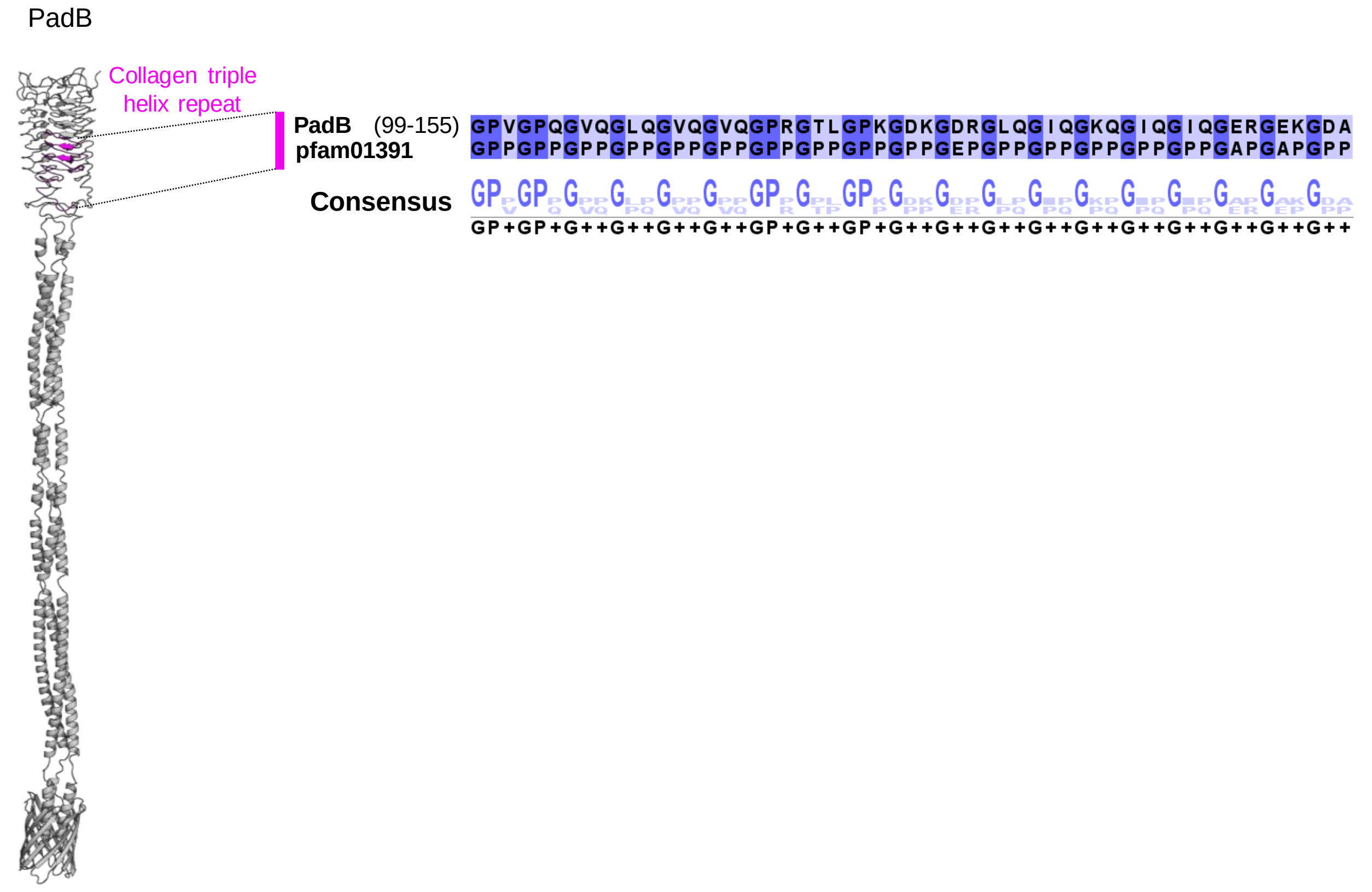

Supplementary Figure S6. **Structural localization and sequence conservation of repetitive regions identified in PadA and PadB.** Predicted structures of PadA and PadB are shown on the left, with the repetitive regions highlighted in red (PadA) and magenta (PadB). For PadA, HHrepID identified eight highly conserved tandem repeats (A1–A8). The multiple sequence alignment reveals strong conservation among the repeat units, particularly within the central core residues. PadB contains a collagen triple-helix repeat region (Pfam01391), displaying the characteristic glycine-rich G–X–Y motif typical of collagen-like domains. Conserved residues are highlighted in blue according to sequence conservation.

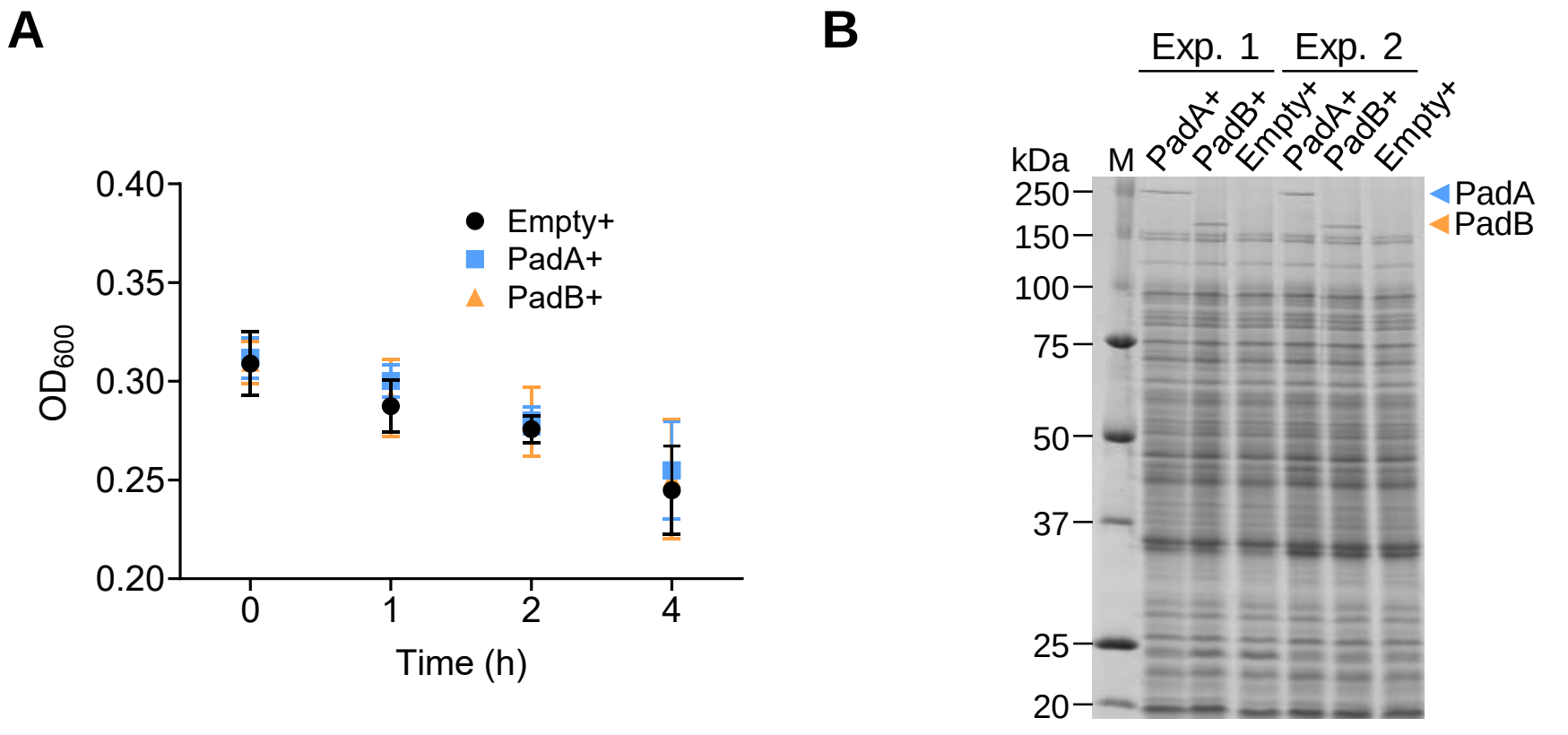

Supplementary Figure S7. Assessment of the role of PadA and PadB in bacterial auto-aggregation. **(A)** Static aggregation assay. Aliquots (5 mL) of MT1415-PadA (PadA+), MT1415-PadB (PadB+) and MT1415-Empty (Empty+) were transferred to 15 mL tubes and incubated in static conditions. Samples were carefully removed from the top of the suspensions at the indicated times to measure the OD_{600}. Results correspond to mean ± standard deviation from two independent experiments, with technical duplicates. **(B)** Analysis of PadA and PadB expression in the bacterial cultures used for the static aggregation assay. Cultures were grown in liquid medium until late exponential phase (OD_{600} of 0.9-1.0). Aliquots were collected, centrifuged, and the pellets subjected to SDS-PAGE in a 12 % polyacrylamide gel, followed by Coomassie Blue staining. M: Molecular weight markers. Blue and orange arrowheads indicate the PadA and PadB bands, respectively.
