# Supplementary material for: Identification and characterization of trimeric autotransporter adhesins from Photobacterium damselae subsp. piscicida
Source: Front Cell Infect Microbiol. 2026 Oct 30;16:1964857. doi: 10.3389/fcimb.2026.1964857 (PMC13759277; doi:10.3389/fcimb.2026.1964857)
Supplement: Supplementary file 2 [file Table1.docx]

| **Nº** | **Strain** | **Origin** | **Year** | **Species** | ***padA*** | ***padB*** | **Nº** | **Strain** | **Origin** | **Year** | **Species** | ***padA*** | ***padB*** |
| --- | --- | --- | --- | --- | --- | --- | --- | --- | --- | --- | --- | --- | --- |
| **1** | SPSA14-1 | Spain | 2014 | *Sparus aurata* | + | + | **26** | SPDL19-2 | Spain | 2019 | *Dicentrarchus labrax* | + | + |
| **2** | SPDL15-1 | Spain | 2015 | *Dicentrarchus labrax* | + | + | **27** | SPDL19-10 | Spain | 2019 | *Dicentrarchus labrax* | + | + |
| **3** | SPDL18-3 | Spain | 2018 | *Dicentrarchus labrax* | - | - | **28** | GRDL19-2 | Greece | 2019 | *Dicentrarchus labrax* | - | - |
| **4** | SPSA16-1 | Spain | 2016 | *Sparus aurata* | + | + | **29** | SPDL19-3 | Spain | 2019 | *Dicentrarchus labrax* | + | + |
| **5** | SPDL17-1 | Spain | 2017 | *Dicentrarchus labrax* | + | + | **30** | SPDL19-4 | Spain | 2019 | *Dicentrarchus labrax* | - | - |
| **6** | SPDL17-2 | Spain | 2017 | *Dicentrarchus labrax* | - | - | **31** | SPDL19-5 | Spain | 2019 | *Dicentrarchus labrax* | - | - |
| **7** | GRDL17-1 | Greece | 2017 | *Dicentrarchus labrax* | - | - | **32** | SPDL19-6 | Spain | 2019 | *Dicentrarchus labrax* | - | - |
| **8** | SPDL17-3 | Spain | 2017 | *Dicentrarchus labrax* | + | + | **33** | SPDL19-7 | Spain | 2019 | *Dicentrarchus labrax* | - | - |
| **9** | SPDL17-4 | Spain | 2017 | *Dicentrarchus labrax* | + | + | **34** | SPDL19-8 | Spain | 2019 | *Dicentrarchus labrax* | + | + |
| **10** | SPDL17-5 | Spain | 2017 | *Dicentrarchus labrax* | - | + | **35** | SPDL19-9 | Spain | 2019 | *Dicentrarchus labrax* | - | + |
| **11** | SPDL18-1 | Spain | 2018 | *Dicentrarchus labrax* | - | - | **36** | GRSA20-1 | Greece | 2020 | *Sparus aurata* | - | - |
| **12** | SPSA18-1 | Spain | 2018 | *Sparus aurata* | + | + | **37** | GRDL20-1 | Greece | 2020 | *Dicentrarchus labrax* | - | - |
| **13** | SPSA18-2 | Spain | 2018 | *Sparus aurata* | - | - | **38** | SPDL20-1 | Spain | 2020 | *Dicentrarchus labrax* | - | - |
| **14** | ITDL18-1 | Italy | 2018 | *Dicentrarchus labrax* | - | - | **39** | SPDL20-2 | Spain | 2020 | *Dicentrarchus labrax* | - | - |
| **15** | SPDL18-2 | Spain | 2018 | *Dicentrarchus labrax* | + | + | **40** | SPDL20-3 | Spain | 2020 | *Dicentrarchus labrax* | - | - |
| **16** | SPDL18-4 | Spain | 2018 | *Dicentrarchus labrax* | - | - | **41** | SPSA20-1 | Spain | 2020 | *Sparus aurata* | - | - |
| **17** | SPDL18-5 | Spain | 2018 | *Dicentrarchus labrax* | - | - | **42** | SPDL20-4 | Spain | 2020 | *Dicentrarchus labrax* | - | - |
| **18** | SPDL18-6 | Spain | 2018 | *Dicentrarchus labrax* | - | - | **43** | SPDL20-5 | Spain | 2020 | *Dicentrarchus labrax* | - | - |
| **19** | SPDL18-7 | Spain | 2018 | *Dicentrarchus labrax* | - | - | **44** | SPDL20-6 | Spain | 2020 | *Dicentrarchus labrax* | - | - |
| **20** | SPDL19-1 | Spain | 2019 | *Dicentrarchus labrax* | - | - | **45** | SPDL20-7 | Spain | 2020 | *Dicentrarchus labrax* | + | + |
| **21** | GRDL19-1 | Greece | 2019 | *Dicentrarchus labrax* | + | + | **46** | SPSA20-2 | Spain | 2020 | *Sparus aurata* | + | + |
| **22** | SPSA19-1 | Spain | 2019 | *Sparus aurata* | - | - | **47** | SPDL20-8 | Spain | 2020 | *Dicentrarchus labrax* | - | - |
| **23** | SPSA19-2 | Spain | 2019 | *Sparus aurata* | + | + | **48** | SPDL20-9 | Spain | 2020 | *Dicentrarchus labrax* | - | - |
| **24** | GRSA19-1 | Greece | 2019 | *Sparus aurata* | + | + | **49** | PTDL20-1 | Portugal | 2020 | *Dicentrarchus labrax* | - | + |
| **25** | ITDL19-1 | Italy | 2019 | *Dicentrarchus labrax* | + | + | **50** | GRDL20-2 | Greece | 2020 | *Dicentrarchus labrax* | - | - |

**Supplementary Table S1**. PCR detection of *padA* and *padB* in *Photobacterium damselae* subsp. *piscicida* field isolates*.*

**Supplementary Table S2**. Oligonucleotides used in this study.

| **Code** | **Purpose** | **Sequence 5’-3’(*)** |
| --- | --- | --- |
| padA_fwd | Detection of *padA* in *Phdp* field isolates | GCCCATGGGCATGCATAAATTTAGATTA |
| padA_rev | Detection of *padA* in *Phdp* field isolates | GCCTCGAGTTGAAACGAATATCCTAT |
| padB_fwd | Detection of *padB* in *Phdp* field isolates | GCCCATGGGCATGAACACTATAGGTAAG |
| padB_rev | Detection of *padB* in *Phdp* field isolates | GCCTCGAGCCAAGTCTTACTAATGCC |
| NcoI_PadA_M1_FW | Cloning of *padA* sequences in pET28a | GCG**CCATGG**GCCATAAATTTAGATTATCAGC |
| XhoI_PadA_Q454_RV | Cloning of *padA* sequences in pET28a | GCG**CTCGAG**TTGAAACGAATATCCTATACC |
| NcoI_PadA_V228_FW | Cloning of *padA* sequences in pET28a | GCG**CCATGG**TCTCGACTGACGCACGTTTTAC |
| XhoI_PadA_V368_RV | Cloning of *padA* sequences in pET28a | GCG**CTCGAG**CACTTTATTGTTTAGAAAAG |
| NcoI_PadB_M1_FW | Cloning of *padB* sequences in pET28a | GC**CCATGG**GCAACACTATAGGTAAGTTTGG |
| XhoI_PadB_W389_RV | Cloning of *padB* sequences in pET28a | GC**CTCGAG**CCAAGTCTTACTAATGCC |
| NcoI_PadB_A28_FW | Cloning of *padB* sequences in pET28a | GC**CCATGG**CCCCACATCGAGATAGTTTAG |
| XhoI_PadB_A206_RV | Cloning of *padB* sequences in pET28a | GC**CTCGAG**TGCAAAGGTACTATCTGTTTGC |
| NcoI_PadB_V172_FW | Cloning of *padB* sequences in pET28a | GC**CCATGG**TGTCTAAAGATAATGCTGATAGC |
| XhoI_PadB_A327_RV | Cloning of *padB* sequences in pET28a | GC**CTCGAG**AGCAAGGGTAGGGATGTTAGCC |
| comp_PadA_fwd | Cloning of *padA* in pMRB24 | GC**GGATCC**CTTCGTTACAGCCATTAG |
| comp_PadA_rev | Cloning of *padA* in pMRB24 | GC**GGATCC**GACAGAACAGATTATAGT |
| comp_PadB_fwd | Cloning of *padB* in pMRB24 | GC**GGATCC**TGGTTTGTCTGGGTGCAT |
| comp_PadB_rev | Cloning of *padB* in pMRB24 | GC**GGATCC**TATTTCACCACTTCACTA |
| pMRB24_check_fwd | Sequencing of pMRB24 insertions | CGCCTGCTATATGCTTGCAT |
| pMRB24_check_rev | Sequencing of pMRB24 insertions | GCTGCTGGGATTACACATGG |

**Supplementary Table S3.** Nucleotide and amino acid sequences of PadA, PadB and PadC from *Photobacterium damselae* subsp. *piscicida* strain GRDL19-1.

| **Protein** | **Nucleotide sequence** | **Amino acid sequence** |
| --- | --- | --- |
| **PadA** | atgcataaatttagattatcagcaattgtagtagctagtgttcttagtacaaatgctttagctgagagtattgatctacctggacgagctcaatctactggcattactggacgtgatggacgtgatggacgtgatggacgtgatggacgtgatggacaattatcaacagaacaattgaagcttattaatggcttaagtgagcgtattaaggctcgcgaagcctttactgacaaatactttgcacaaaccgattatattcttggcgaaaacttggcaggaatagtaaataatggtcgattaatctataacttagatcttatcaaagctgatgcagtctcgactgacgcacgttttacagaagctcgtgaaaatattggcagtaacacgactaaaatagcaacaaacacaaaatcaattaataacttaagtgagcgtattgaggctggcgaagctttaactgacacacgttttacagaggctcgtgaaaatattggcagtaacacgactaaaatagcaacaaacacaacatcaattaatagcttaagtgagcgtattaaggctggcgaagccttaactgacaaatactttgcacaaacccatgatattcttgacaaaaacttggcagaaacagcaaataatagtcgattaatctataacttagatcttatcaaagctgatgcagtctcgactgacgcacgttttacagaagctcgtgaaaatattggcagtaacacgactaaaatagcaacaaacacaaaatcaattaatagcttaagtgagcgtattgaggctggcgaagctttaactgacacacgttttacagaaactcgtaaaaatattagcagtaacacgactaaaatagcaacaaacacaaaatcaattaatagcttaagtgagcgtattgaggctggcgaagctttaactgacacacgttttacagaaactcgtaaaaatattagcagtaacatgactaaaatagcaacaaatacaaaaaatgtagctaataacgcaaaagcaattatatctaattcaaatacgctggagagatatgggaagcagttaaatgaacacgacgagaaaataacttttctaaacaataaagtggaccgaaacactggattgataaaagaaaatacttatcgcataaataagttagagcacggtatgtcgttaatggctgcaatgtctaatttaacacaagatgatgtaacgatggctctcggctactataatggtaatacagctctagcaatgggaataaattatgcgttaatgcctaatatgaatgttaaatttagtgctgctgctgacacggaattcacacaaacatctcttggtgttggtataggatattcgtttcaataa | MHKFRLSAIVVASVLSTNALAESIDLPGRAQSTGITGRDGRDGRDGRDGRDGQLSTEQLKLINGLSERIKAREAFTDKYFAQTDYILGENLAGIVNNGRLIYNLDLIKADAVSTDARFTEARENIGSNTTKIATNTKSINNLSERIEAGEALTDTRFTEARENIGSNTTKIATNTTSINSLSERIKAGEALTDKYFAQTHDILDKNLAETANNSRLIYNLDLIKADAVSTDARFTEARENIGSNTTKIATNTKSINSLSERIEAGEALTDTRFTETRKNISSNTTKIATNTKSINSLSERIEAGEALTDTRFTETRKNISSNMTKIATNTKNVANNAKAIISNSNTLERYGKQLNEHDEKITFLNNKVDRNTGLIKENTYRINKLEHGMSLMAAMSNLTQDDVTMALGYYNGNTALAMGINYALMPNMNVKFSAAADTEFTQTSLGVGIGYSFQ |
| **PadB** | atgaacactataggtaagtttggtttatctgcattagcaatagcagtggtgactggtgtttttagtgcaaatgctttagcagccccacatcgagatagtttagaaagctcattagtaagtgacgactcaatcctttctttaccaacagtttcttacgcagatcgtctaaacggtgtaaccaatcaccgctccccaatgatggtagcagcccctactgacacaggtgtccgttctcatggacatcttgttggttatgaagtaggagacggtttagatatctttcctagtactcgtggccctgtcggtcctcaaggcgttcaaggccttcaaggcgttcaaggcgttcaaggccctagaggaactattggtcccaaaggcgacaagggtgatcgtggtcttcaaggcatccaaggtaagcaaggtattcagggaatccaaggcgaacgtggtgaaaaaggtgatgctggcggtttatctggagagcaagaacaacgcctagctatgattgaagaagtgtctaaagataatgctgatagcattacccaaaacactactagcattcacagcttaaacactactcttgctggcgtgaaaaagcaaacagatagtacctttgcatctgtgggtgctcagttagatgatatcaataacacacaaactgctactgataagaaagtaacgaataatagtgaacgtctcagtatcgcagaacagcaaaatcaaacgatgggtaagacggttcaagaaaataaaaatcgctccctagttaacgaagaacgctctctaaataatgagtatcgttctgttcataactctaacaagatcgctgacaactccaagcgtcttgatctgagtgaaaagtggcagaagatggcagatcagcgtctttctgacctcgaaggtgatgtacgtcataaccgtaaagttgctgcacgtggtgtggctggcgtagctgctatggctaacatccctacccttgctattcctggttctactagttttggtgctggtatcggccattatgatagtgagtctgcattagctgtaggtgtaagccattactttgataatggtgttgcagtgaaaggttctgtaggtttcacatctggccagagcatcttaggcgctggcattagtaagacttggtaa | MNTIGKFGLSALAIAVVTGVFSANALAAPHRDSLESSLVSDDSILSLPTVSYADRLNGVTNHRSPMMVAAPTDTGVRSHGHLVGYEVGDGLDIFPSTRGPVGPQGVQGLQGVQGVQGPRGTIGPKGDKGDRGLQGIQGKQGIQGIQGERGEKGDAGGLSGEQEQRLAMIEEVSKDNADSITQNTTSIHSLNTTLAGVKKQTDSTFASVGAQLDDINNTQTATDKKVTNNSERLSIAEQQNQTMGKTVQENKNRSLVNEERSLNNEYRSVHNSNKIADNSKRLDLSEKWQKMADQRLSDLEGDVRHNRKVAARGVAGVAAMANIPTLAIPGSTSFGAGIGHYDSESALAVGVSHYFDNGVAVKGSVGFTSGQSILGAGISKTW |
| **PadC** | ttggagtcaccacctagttatgccgctcaaaacaaaagtaatattattatcgataagccaaatgctaccgatccaatcgtaaatggacaaaactatggtaccaatccaattataggtactgtacctaccttttctggatcaaataacgttatcttaggctcaggtaatacagctacgttaaattctacggccatcggagaaaatacaacagccaatgatgcttcaactgcggttggtacagcatcttttgctcaaggaaaaagtttagccattggtacacaatcatattcaacaaatggaagtattgccattggcagccaatcggtatcgactgatggtcaagtctctgtcggtagccagtataagcaacgccgtattgaaaatgttgcggcaggtattgcatcaaatgatgcagttaatgtcagccaattaaataataaaataacgccactacaaaaaaatattgctgataataccaattcaattaatgagacacgtacgacaaccattgaaaatacaaatagtatcaatcagttaagtaacatcgtaaataaccatacaactgatatcaataataattcgaatcaaataactactaatacatcgaatattaagaccaatgctcatcatctcgacactattcaagcgataacgaaaaacgatcaaaaatctatacaaaaaaatgaaaaatcaatcagctcaaatacaacagcaatccaatctaatcagcaaggtatagccacaaacagcgctcgaattgatgatttaaataacaattatcgtgaatatgtcaataataagtttgatcaacttcaaggagacattaaacaggttagaaagcatgctgatgctggtattgcttcagcgatggcggtggtgactccaattgatttattaatggtaaaattcacgttctataaacgttccaataaccttatcgtgcatctcttcgcttttcgaaaaaccaatggtttttcttactaa | MESPPSYAAQNKSNIIIDKPNATDPIVNGQNYGTNPIIGTVPTFSGSNNVILGSGNTATLNSTAIGENTTANDASTAVGTASFAQGKSLAIGTQSYSTNGSIAIGSQSVSTDGQVSVGSQYKQRRIENVAAGIASNDAVNVSQLNNKITPLQKNIADNTNSINETRTTTIENTNSINQLSNIVNNHTTDINNNSNQITTNTSNIKTNAHHLDTIQAITKNDQKSIQKNEKSISSNTTAIQSNQQGIATNSARIDDLNNNYREYVNNKFDQLQGDIKQVRKHADAGIASAMAVVTPIDLLMVKFTFYKRSNNLIVHLFAFRKTNGFSY |
|  |  |  |

**Supplementary Table S4.** Presence and genomic location of *padA-* and *padB-*related sequences, and amino acid sequence identity of the encoded proteins, among *Photobacterium damselae* subsp. *piscicida* and *P. damselae* subsp. *damselae* genomes analysed in this study. Sequences were identified by BLASTp using the corresponding PadA and PadB sequences from strain GRDL19-1 as queries. The comparative analysis included all *P. damselae* genomes available in the NCBI RefSeq database (accessed July 2026) together with the four European *Phdp* genomes generated in this study. Percentage identity (% ID) is relative to the corresponding protein from GRDL19-1.

| **Strain designation** | **Origin/Year** | **Host** | **PadA** (% ID) | **PadB** (% ID) | **Plasmid/Contig** | **BioSample ID/NCBI RefSeq assembly** | **Ref.** |
| --- | --- | --- | --- | --- | --- | --- | --- |
| ***Photobacterium damselae* subsp. *piscicida*** | | | | | | | |
| GRDL19-1 | Greece/2019 | *Dicentrarchus labrax* | n.a.** | n.a.** | pPHDP170 | SAMN61872470 | This study |
| ITDL19-1 | Italy/2019 | *Dicentrarchus labrax* | n.a.** (100%) | n.a.** (100%) | pPHDP170 | SAMN61872471 | This study |
| SPDL19-3 | Spain/2019 | *Dicentrarchus labrax* | n.a.** (100%) | n.a.** (100 %) | pPHDP170 | SAMN61872472 | This study |
| SPSA19-2 | Spain/2019 | *Sparus aurata* | n.a.** (100%) | n.a.** (100 %) | pPHDP170 | SAMN61872473 | This study |
| J23.1 | Spain/2021 | *Dicentrarchus labrax* | WP_491341495.1 (100%) | WP_445324264.1 (100%) | pPHDPT3 | GCF_058273185.1 | - |
| AS-16-0555-7 | Australia/2016 | *Seriola lalandi* | WP_191169544.1 (96.3%) | WP_191169556.1 (99%) | Unnamed1 | GCF_014775695.1 | (Baseggio et *al.*, 2021) |
| QMA0776 | Australia/2022 | *Seriola lalandi* | WP_191169544.1 (96.3%) | WP_191169556.1 (99%) | pPHDPT3_Stable | GCF_053202905.1 | (Rudenko et *al.*, 2026) |
| Y25OFS1 | South Korea/2025 | *Oplegnathus fasciatus* | WP_191169544.1 (96.3%) | WP_191169556.1 (99%) | Unnamed1 | GCF_058410825.1 | - |
| OT-51443 | Japan/1995 | *Seriola quinqueradiata* | WP_086959556.1 (96%) | - | Contig_NZ_BDMQ01000003.1 | GCF_002157645.1 | (Aoki et al., 2017) |
| 91-197 | USA/2003 | *Morone chrysops X Morone saxatilis* | WP_096498264.1 (95.8%) | - | p91-197-2 | GCF_002356235.1 | (Teru et al., 2017) |
| PP3 | Japan/unknown | *Seriola quinqueradiata* | - | WP_191169556.1 (99%) | pPP3_T3SS | GCF_004683985.2 | (Abushattal et al., 2019) |
| MT1415 | Italy/unknown | *Dicentrarchus labrax* | - | - | - | GCF_005048985.1 | - |
| ***Photobacterium damselae* subsp. *damselae*** | | | | | | | |
| ATCC 33539* | USA/1982 | *Pomacentridae* | WP_211315314.1 (91.1%) WP_146154901.1 (92.5%) | WP_005306612.1 (60 %) | Contig_NZ_PYOG01000081.1 Contig_NZ_PYOG01000056.1 Contig_NZ_PYOG01000046.1 | GCF_003026815.1 | - |
| CIP 102761* | Unknown/unknown | *Pomacentridae* | WP_005306645.1 (91%) | WP_005306612.1 (60 %) | Contig_NZ_ADBS01000003.1 | GCF_000176795.1 | - |
| NCTC11647* | Unknown/1984 | *Pomacentridae* | WP_005306645.1 (93.1%) | WP_005306612.1 (60 %) | Contig_NZ_UATL01000006.1 | GCF_900454585.1 | - |

*ATCC 33539, CIP 102761 and NCTC 11647 correspond to different genome assemblies of the same *Phdd* type strain (Sapio et al., 2026). In the CIP 102761 assembly, contig NZ_ADBS01000003.1 corresponds to the 203-kb plasmid pPHDD203 (ADBS01000003.1) (Vences et al., 2017). n.a.** The genome sequences of *Photobacterium damselae* subsp. *piscicida* strains GRDL19-1, ITDL19-1, SPDL19-3 and SPSA19-2 have been submitted to GenBank under BioProject accession number PRJNA1499273.

**References:**

Abushattal, S., Vences, A., Dos Santos, N.M.S., do Vale, A., and Osorio, C.R. (2019). Draft Genome Sequences of *Photobacterium damselae* subsp. *piscicida* SNW-8.1 and PP3, Two Fish-Isolated Strains Containing a Type III Secretion System. *Microbiol Resour Announc* 8(21). doi: 10.1128/MRA.00426-19.

Aoki, T., Teru, Y., Morimoto, N., Kono, T., Sakai, M., Takano, T., et al. (2017). Complete Genome Sequence of *Photobacterium damselae* subsp. *piscicida* Strain OT-51443 Isolated from Yellowtail (*Seriola quinqueradiata*) in Japan. *Genome Announc* 5(21). doi: 10.1128/genomeA.00404-17.

Baseggio, L., Rudenko, O., Buller, N., Landos, M., Englestadter, J., and Barnes, A.C. (2021). Complete, closed and curated genome sequences of *Photobacterium damselae* subsp. *piscicida* isolates from Australia indicate mobilome-driven localized evolution and novel pathogenicity determinants. *Microb Genom* 7(4). doi: 10.1099/mgen.0.000562.

Rudenko, O., Baseggio, L., and Barnes, A.C. (2026). The Type III Secretion System plasmid pPHDPT3 of *Photobacterium damselae* subsp. *piscicida* is stable in Australian isolates due to conserved non-repetitive genomic architecture. *FEMS Microbiol Lett* 373. doi: 10.1093/femsle/fnag074.

Sapio, M., Mellace, M., Matanza, X.M., Ambrosio, R.L., Pace, A., Tilocca, B., et al. (2026). *Photobacterium damselae* subsp. *damselae* at the animal-human interface: ecology, pathogenicity, and genomic gaps in a changing climate. *Front Microbiol* 17, 1901411. doi: 10.3389/fmicb.2026.1901411.

Teru, Y., Hikima, J.I., Kono, T., Sakai, M., Takano, T., Hawke, J.P., et al. (2017). Whole-Genome Sequence of *Photobacterium damselae* subsp. *piscicida* Strain 91-197, Isolated from Hybrid Striped Bass (*Morone* sp.) in the United States. *Genome Announc* 5(29). doi: 10.1128/genomeA.00600-17.

Vences, A., Rivas, A.J., Lemos, M.L., Husmann, M., and Osorio, C.R. (2017). Chromosome-Encoded Hemolysin, Phospholipase, and Collagenase in Plasmidless Isolates of *Photobacterium damselae* subsp. *damselae* Contribute to Virulence for Fish. *Appl Environ Microbiol* 83(11). doi: 10.1128/AEM.00401-17.

**Supplementary Table S5.** Presence and genomic location of *padC*-related sequences, and amino acid sequence identity of the encoded PadC proteins, among *Photobacterium damselae* subsp. *piscicida* and *P.* *damselae* subsp. *damselae* genomes analyzed in this study. PadC-related proteins were identified by BLASTp using the PadC protein sequence from strain GRDL19-1 as query. The comparative analysis included all *P. damselae* genomes available in the NCBI RefSeq database (accessed July 2026) together with the four European *Phdp* genomes generated in this study. Percentage identity (% ID) is relative to the PadC protein from strain GRDL19-1.

| **Strain designation** | **Origin/year** | **Host** | **PadC** (% ID) | **Localization** | **BioSample ID/NCBI RefSeq assembly** |
| --- | --- | --- | --- | --- | --- |
| ***Photobacterium damselae* subsp. *piscicida* European strains** | | | | | |
| GRDL19-1 | Greece/2019 | *Dicentrarchus labrax* | n.a.* | Chromosome 2 | SAMN61872470 |
| ITDL19-1 | Italy/2019 | *Dicentrarchus labrax* | n.a.* (100 %) | Chromosome 2 | SAMN61872471 |
| SPDL19-3 | Spain/2019 | *Dicentrarchus labrax* | n.a.* (100 %) | Chromosome 2 | SAMN61872472 |
| SPSA19-2 | Spain/2019 | *Sparus aurata* | n.a.* (100 %) | Chromosome 2 | SAMN61872473 |
| GalP1 | Greece/2021 | *Sparus aurata* | WP_475840424.1 (100%) | Chromosome 2 | GCF_056234105.1 |
| J23.1 | Spain/2021 | *Dicentrarchus labrax* |  | Chromosome 2 | GCF_058273185.1 |
| ML9 | Greece/2023 | *Dicentrarchus labrax* |  | Chromosome 2 | GCF_056234175.1 |
| MM52 | Greece/2021 | *Dicentrarchus labrax* |  | Chromosome 2 | GCF_053339145.1 |
| MT1415 | Italy/unknown | *Dicentrarchus labrax* | TJZ95645.1 (partial seq; 100%) | Contig_000146 | GCF_005048985.1 |
| ***Photobacterium damselae* subsp. *piscicida* Asia-Pacific and USA strains** | | | | | |
| AS-16-0555-7 | Australia/2016 | *Seriola lalandi* | WP_086959024.1 (97.5%) | Chromosome 2 | GCF_014775695.1 |
| QMA0776 | Australia/2022 | *Seriola lalandi* |  | Chromosome 2 | GCF_053202905.1 |
| OT-51443 | Japan/1995 | *Seriola quinqueradiata* |  | Contig_NZ_BDMQ01000002.1 | GCF_002157645.1 |
| PP3 | Japan/unknown | *Seriola quinqueradiata* |  | Chromosome 2 | GCF_004683985.2 |
| Y25OFS1 | South Korea/2025 | *Oplegnathus fasciatus* |  | Chromosome 2 | GCF_058410825.1 |
| 91-197 | USA/2003 | *Morone chrysops x Morone saxatilis* |  | Chromosome 2 | GCF_002356235.1 |
| ***Photobacterium damselae* subsp. *damselae*** | | | | | |
| 111bp-OG15A | Turkey/2011 | *Dicentrarchus labrax* | WP_138241159.1 (89.2%) | Contig_000124 | GCF_005819825.1 |
| 144bp-OG3 | Turkey/2011 | *Dicentrarchus labrax* | WP_106262101.1 (89.2%) | Contig_000162 | GCF_005819835.1 |
| 164dp-OG2 | Turkey/2011 | *Dicentrarchus labrax* |  | Contig_000097 | GCF_006297395.2 |
| 206328-2 | Denmark/2009 | *Oncorhynchus mykiss* |  | Contig_000117 | GCF_003004595.1 |
| 70dps-OG12 | Turkey/2011 | *Dicentrarchus labrax* | WP_138241159.1 (89.2%) | Contig_000036 | GCF_005819805.1 |
| AS-15-3942-7 | Australia/2015 | *Seriola lalandi* | WP_237035736.1 (89.2%) | Chromosome 2 | GCF_021768405.1 |
| AS-15-3942-8 | Australia/2015 | *Seriola lalandi* | WP_237030430.1 (88.9%) | Chromosome 2 | GCF_021768385.1 |
| AS-15-3942-9 | Australia/2015 | *Seriola lalandi* | WP_237035736.1 (89.2%) | Chromosome 2 | GCF_021768365.1 |
| BT-6 | USA/2008 | nd | WP_065171361.1 (88.2%) | Contig_CFSAN065500_1 | GCF_003025675.1 |
| BV30 | China/2022 | *Shrimp* | WP_106262101.1 (89.2%) | Chromosome 2 | GCF_054486525.1 |
| CSP DAM1 | Australia/2010 | *Seriola lalandi* | WP_237034155.1 (89.2%) | Chromosome 2 | GCF_021766015.1 |
| CSP DAM2 | Australia/2010 | *Seriola lalandi* |  | Chromosome 2 | GCF_021765875.1 |
| DNB23_24_2 | USA/2023 | *Crassostrea virginica* | WP_446513905.1 (89.5%) | Contig_NZ_JBNCDP010000007.1 | GCF_052878375.1 |
| LD-07 | Spain/1991 | *Sparus aurata* | WP_068968697.1 (88.5%) | Contig_2 | GCF_001707815.1 |
| M13-17570 | Australia/2013 | *Magallana gigas* | WP_239970746.1 (88.9%) | Contig_NZ_JAKEVP010000003.1 | GCF_022398535.1 |
| M270202 | Mexico/2022 | *Penaeus vannamei* | WP_331434951.1 (84.3%) | Contig_NZ_JATAAZ010000711.1  Contig_NZ_JATAAZ010000075.1 | GCF_036533535.1 |
| PBN23_20_6 | USA/2023 | *Crassostrea virginica* | WP_446513905.1 (89.5%) | Contig_NZ_JBNCCU010000004.1 | GCF_052877475.1 |
| Pdd1411 | China/2014 | nd | WP_284648379.1 (88.5%) | Chromosome 2 | GCF_030168855.1 |
| Ph4 | China/2020 | *Larimichthys crocea* | WP_238070830.1 (88.9%) | Contig_NZ_JADQAR010000004.1 | GCF_022129505.1 |
| PNB23_20_2 | USA/2023 | *Crassostrea virginica* | WP_446513905.1 (89.5%) | Contig_NZ_JBNCCR010000009.1 | GCF_052877355.1 |
| PNB23_20_8 | USA/2023 | *Crassostrea virginica* |  | Contig_NZ_JBNCCP010000012.1 | GCF_052877275.1 |
| PNB23_20_10 | USA/2023 | *Crassostrea virginica* |  | Contig_NZ_JBNCCS010000010.1 | GCF_052877375.1 |

n.a.* The genome sequences of *Photobacterium damselae* subsp. *piscicida* strains GRDL19-1, ITDL19-1, SPDL19-3 and SPSA19-2 have been submitted to GenBank under BioProject accession number PRJNA1499273.

**Supplementary Table S6**. Predicted PadC protein variants identified in *Photobacterium damselae* strains

| ***P. damselae* group** | **Representative strains** | **length (aa)** | **Predicted N-terminal signal peptide*** | **C-terminal region** | **YadA-family fragment**** |
| --- | --- | --- | --- | --- | --- |
|  | | | | | |
| European *Phdp* | GRDL19-1, ITDL19-1, SPDL19-3, SPSA19-2, GalP1, J23.1, ML9, MM52 | 327 | Absent | Shorter *Phdp*-type C-terminal region; no unambiguous complete β-barrel-like structure predicted | Present |
| Asia-Pacific/USA *Phdp* | AS-16-0555-7, QMA0776, OT-51443, PP3, Y25OFS1, 91-197 | 338 | Present | Shorter *Phdp*-type C-terminal region; no unambiguous complete β-barrel-like structure predicted | Absent |
| *Phdd* | 111bp-OG15A, 144bp-OG3, 164dp-OG2, 206328-2, 70dps-OG12, AS-15-3942-7, AS-15-3942-8, AS-15-3942-9, BT-6, BV30, CSP DAM1, CSP DAM2, DNB23_24_2, LD-07, M13-17570, M270202, PBN23_20_6, Pdd1411, Ph4, PNB23_20_2, PNB23_20_8, PNB23_20_10 | 369 | Present | Extended *Phdd*-type C-terminal region; well-defined β-barrel-like structure predicted with high confidence | Absent |
|  |  |  |  |  |  |

* Signal peptides were predicted with SignalP - 6.0 server (https://services.healthtech.dtu.dk/services/SignalP-6.0/); ** A separate 73-aa YadA C-terminal domain-containing ORF was identified in the European *Phdp* strains. The C-terminal 68 residues of its predicted product are identical to the corresponding terminal region of *Phdd* PadC
